# XPA lactylation is required for UV-induced DNA damage repair

**DOI:** 10.1093/nar/gkag947

**Published:** 2026-10-07

**Authors:** Jie Yang, Xin Ding, Zhikai Zeng, Hui Yin, Yiming He, Mingpeng Jin, Bingsong Huang, Zhe Wang, Xiaoning Yang, Rui Li, Qianwen Wang, Xuan Zhou, Yifei Song, Yu Li, Xuanhe Wang, Wen Zheng, Lu Lu, Qian Zhu, Jiaqi Liu, Zhihua Kang, Lei Li, Yunhui Li, Chengyuan Wang, Jian Yuan, Yuping Chen, Jinhuan Wu, Bentong Yu

**Affiliations:** Jiangxi Provincial Key Laboratory of Respiratory Diseases; Jiangxi Institute of Respiratory Diseases; Jiangxi Clinical Research Center for Respiratory Diseases; Department of Thoracic Surgery, The First Affiliated Hospital, Jiangxi Medical College, Nanchang University, Nanchang 330006, China; China-Japan Friendship Jiangxi Hospital; National Regional Center for Respiratory Medicine, Nanchang 330000, China; Cancer Center, Tongji University School of Medicine, Shanghai 200331, China; State Key Laboratory of Cardiovascular Diseases and Medical Innovation Center, Shanghai East Hospital, School of Medicine, Tongji University, Shanghai 200120, China; Jiangxi Provincial Key Laboratory of Respiratory Diseases; Jiangxi Institute of Respiratory Diseases; Jiangxi Clinical Research Center for Respiratory Diseases; Department of Thoracic Surgery, The First Affiliated Hospital, Jiangxi Medical College, Nanchang University, Nanchang 330006, China; China-Japan Friendship Jiangxi Hospital; National Regional Center for Respiratory Medicine, Nanchang 330000, China; Department of Thoracic Surgery, The First Affiliated Hospital of Shaoyang University, Shaoyang 422000, China; Cancer Center, Tongji University School of Medicine, Shanghai 200331, China; Shanghai Pulmonary Hospital, Tongji University School of Medicine, Shanghai 200433, China; Cancer Center, Tongji University School of Medicine, Shanghai 200331, China; Translational Research Institute of Brain and Brain-Like Intelligence, Shanghai Fourth People’s Hospital, School of Medicine, Tongji University, Shanghai 200434, China; Cancer Center, Tongji University School of Medicine, Shanghai 200331, China; State Key Laboratory of Cardiovascular Diseases and Medical Innovation Center, Shanghai East Hospital, School of Medicine, Tongji University, Shanghai 200120, China; Cancer Center, Tongji University School of Medicine, Shanghai 200331, China; State Key Laboratory of Cardiovascular Diseases and Medical Innovation Center, Shanghai East Hospital, School of Medicine, Tongji University, Shanghai 200120, China; Institute of Materia Medica, Chinese Academy of Medical Sciences & Peking Union Medical College, Beijing 100050, China; Cancer Center, Tongji University School of Medicine, Shanghai 200331, China; State Key Laboratory of Cardiovascular Diseases and Medical Innovation Center, Shanghai East Hospital, School of Medicine, Tongji University, Shanghai 200120, China; Cancer Center, Tongji University School of Medicine, Shanghai 200331, China; Translational Research Institute of Brain and Brain-Like Intelligence, Shanghai Fourth People’s Hospital, School of Medicine, Tongji University, Shanghai 200434, China; Department of Radiation Oncology, Ruijin Hospital, Shanghai Jiao Tong University School of Medicine, Shanghai 200025, China; Cancer Center, Tongji University School of Medicine, Shanghai 200331, China; State Key Laboratory of Cardiovascular Diseases and Medical Innovation Center, Shanghai East Hospital, School of Medicine, Tongji University, Shanghai 200120, China; Cancer Center, Tongji University School of Medicine, Shanghai 200331, China; State Key Laboratory of Cardiovascular Diseases and Medical Innovation Center, Shanghai East Hospital, School of Medicine, Tongji University, Shanghai 200120, China; Cancer Center, Tongji University School of Medicine, Shanghai 200331, China; Shanghai Pulmonary Hospital, Tongji University School of Medicine, Shanghai 200433, China; Medical and Radiation Oncology, Department of the Second Affiliated Hospital of Wenzhou Medical University, Wenzhou 325000, China; Cancer Center, Tongji University School of Medicine, Shanghai 200331, China; State Key Laboratory of Cardiovascular Diseases and Medical Innovation Center, Shanghai East Hospital, School of Medicine, Tongji University, Shanghai 200120, China; Department of Radiation Oncology, Ruijin Hospital, Shanghai Jiao Tong University School of Medicine, Shanghai 200025, China; Department of Burn, Ruijin Hospital, Shanghai Jiao Tong University School of Medicine, Shanghai 200025, China; Laboratory Medicine, Shanghai East Hospital, Tongji University School of Medicine, Shanghai 200025, China; Cancer Center, Tongji University School of Medicine, Shanghai 200331, China; State Key Laboratory of Cardiovascular Diseases and Medical Innovation Center, Shanghai East Hospital, School of Medicine, Tongji University, Shanghai 200120, China; Cancer Center, Tongji University School of Medicine, Shanghai 200331, China; State Key Laboratory of Cardiovascular Diseases and Medical Innovation Center, Shanghai East Hospital, School of Medicine, Tongji University, Shanghai 200120, China; Shanghai Institute of Materia Medica, Chinese Academy of Sciences, Shanghai 200032, China; Cancer Center, Tongji University School of Medicine, Shanghai 200331, China; Translational Research Institute of Brain and Brain-Like Intelligence, Shanghai Fourth People’s Hospital, School of Medicine, Tongji University, Shanghai 200434, China; Cancer Center, Tongji University School of Medicine, Shanghai 200331, China; Translational Research Institute of Brain and Brain-Like Intelligence, Shanghai Fourth People’s Hospital, School of Medicine, Tongji University, Shanghai 200434, China; Cancer Center, Tongji University School of Medicine, Shanghai 200331, China; Putuo People’s Hospital, School of Medicine, Tongji University, Shanghai 200060, China; Jiangxi Provincial Key Laboratory of Respiratory Diseases; Jiangxi Institute of Respiratory Diseases; Jiangxi Clinical Research Center for Respiratory Diseases; Department of Thoracic Surgery, The First Affiliated Hospital, Jiangxi Medical College, Nanchang University, Nanchang 330006, China; China-Japan Friendship Jiangxi Hospital; National Regional Center for Respiratory Medicine, Nanchang 330000, China

## Abstract

Xeroderma pigmentosum group A (XPA) serves as a core nucleotide excision repair (NER) factor essential for the repair process triggered by UV irradiation. Lactylation, a newly identified post-translational modification, plays a key role in tumorigenesis and cancer progression. Herein, we discovered that XPA was lactylated in the DNA-binding domain by the acyl-transferase KAT5. In addition, UV irradiation promoted XPA lactylation, thereby increasing the chromatin recruitment of XPA and NER. However, the mutation of XPA to a lactylation-deficient form impeded XPA chromatin recruitment under UV irradiation, impaired NER, and rendered cancer cells hypersensitive to UV irradiation. Our data demonstrate that the lactylation of XPA plays a crucial role in NER and reveals the link between metabolic reprogramming and DNA damage repair induced by UV irradiation.

## Graphical Abstract

**Graphical Abstract:**
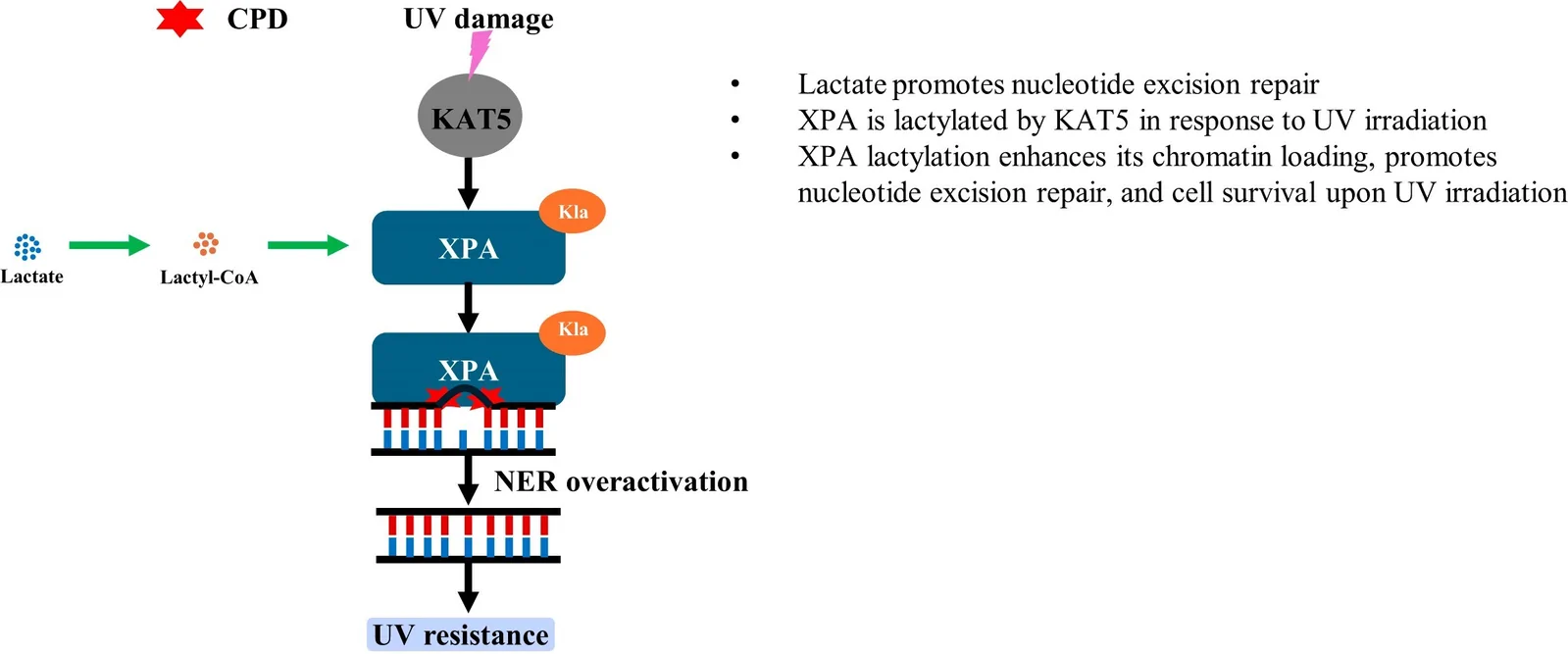

## Introduction

Metabolic reprogramming is a prominent feature of cancer. Notably, even under aerobic conditions, the majority of cancer cells prefer to metabolize glucose through glycolysis, leading to the generation of a significant amount of lactate. This phenomenon, i.e. the Warburg effect, was first described by Otto Heinrich Warburg in 1923 [1] and is characterized by high levels of glycolysis and low levels of oxidative phosphorylation [2]. In 2019, Zhao *et al*. discovered a novel post-translational modification of histone lysine residues with a mass shift of 72.021 Da: lactylation [3]. Glucose, rotenone, and hypoxia have been found to increase lactylation, whereas inhibiting glycolysis or knocking out lactate dehydrogenase has been shown to have the opposite effect. An increasing number of studies have demonstrated that lactylation plays a crucial role in tumorigenesis and progression. p53 lactylation mediated by AARS1 and eEF1A2 lactylated by KAT8 can promote tumorigenesis [4, 5]. AARS2-mediated cGAS lactylation leads to immune escape [6]. Moreover, the inhibition of MRE11 lactylation by a specific peptide, a pivotal homologous recombination (HR) protein, impedes DNA end resection and HR, ultimately resulting in increased tumor cell chemosensitivity [7]. Lactylation is closely related to the development, progression, and chemoradiotherapy resistance of tumors. Nevertheless, the detailed underlying mechanism remains incompletely understood.

The integrity of genomic DNA is constantly threatened by both endogenous factors (e.g. replication stress and reactive oxygen species) and exogenous factors [e.g. ultraviolet (UV), chemical drugs, and ionizing radiation) [8, 9]. Cells incur ~10^5^ instances of DNA damage daily [10]; thus, repairing this damage in an efficient and timely manner is necessary to maintain genome integrity. Nucleotide excision repair (NER) is the most ubiquitous DNA damage repair pathway capable of repairing a variety of DNA helix distortions. The DNA damage substrates recognized by NER include cyclobutane pyrimidine dimers (CPDs) and pyrimidine-pyrimidone-(6-4)-photoproducts (6-4PPs) induced by UV irradiation, interstrand crosslinks, and large base adducts caused by chemical drugs or certain carcinogens [11]. NER can be divided into global genome NER and transcription-coupled NER based on the lesion recognized. The NER pathway, involving over 30 proteins, includes DNA lesion recognition/verification, helix unwinding, dual incision/excision, gap-filling synthesis, and ligation [12]. Furthermore, NER is the only DNA damage repair pathway that removes UV-induced DNA damage, thereby protecting the skin from UV radiation damage, maintaining genomic stability, and preventing tumorigenesis.

Xeroderma pigmentosum group A (XPA), which contains 273 amino acids, is a key repair protein that can bind to DNA in the NER process; it plays a crucial role in the recognition of DNA damage and the recruitment of other NER repair proteins [13]. During NER, XPA can be recruited to the site of DNA damage via several mechanisms and functions as a scaffold protein, serving as a bridge to transmit damage signals and recruit downstream repair factors to promote NER [14]. The deregulation of XPA is closely associated with tumorigenesis and cancer progression [15]. In nasopharyngeal carcinoma, EZH2 suppresses XPA transcription by facilitating the trimethylation of H3K27 in the XPA promoter region, which consequently results in increased sensitivity to UV and cisplatin [16]. In addition, UV radiation enhances the SIRT1-XPA interaction, decreases XPA acetylation, XPA–RPA32 binding, and NER activity, thereby promoting tumor cell UV sensitivity [17]. Moreover, under UV irradiation, PARP1-mediated XPA PARylation promotes XPA recruitment to DNA damage sites and increases NER activity [18]. However, the regulatory mechanism of XPA has yet to be elucidated, and it is unclear whether tumor metabolism, especially lactate metabolism, can regulate XPA function and affect NER repair.

Herein, we discover that protein lactylation regulates UV-induced DNA damage. XPA is lactylated by KAT5 at lysines located in its DNA-binding domain. This lactylation enhances the chromatin loading of XPA and NER under UV-induced damage, thereby decreasing the UV sensitivity of tumor cells. Conversely, the loss of XPA lactylation compromises NER and results in cell hypersensitivity toward UV irradiation. Our results indicate that lysine lactylation is a crucial post-translational modification that regulates XPA protein function and NER in response to genotoxic stress, suggesting potential therapeutic strategies to prevent tumorigenesis and overcome chemotherapy resistance in cancer treatment.

## Materials and methods

### Cell culture, DNA transfection, virus packaging, and lentiviral infection

HEK293T, HeLa, A549, A375, and MDA-MB-231 cells were purchased from ATCC and were fed with Dulbecco’s modified Eagle’s medium supplemented with 10% fetal bovine serum and 1% penicillin and streptomycin in a 37°C incubator with 5% CO_2_. Cell transfection was performed by utilizing Polyetherimide or Lipofectamine 2000 (supplied by Thermo Fisher Scientific). Lentiviral vectors and packaging plasmids (specifically, pMD2G and pSPAX2) were co-transfected, followed by the collection of viral supernatants at 24 and 48 h after transfection. The harvested lentiviruses were subsequently infected into the cells for additional experiments, with the aid of 8 µg/ml polybrene to boost infection efficiency. To obtain stable cell lines, selection was carried out using a medium supplemented with 2 µg/ml puromycin. The stable cells were lysed and blotted with indicated antibodies.

### Antibodies

The following antibodies were used in this study: anti-GAPDH (Proteintech: 60004-1-lg, 1:3000), anti-Flag (Proteintech: 20543-1-AP, 1:2000), anti-HA (Cell Signaling Technology: no.3724, 1:2000), anti-CPD (MBL International Corporation: NMDND-001, 1:600), anti-RPA32 (Santa Cruz: sc-56770, 1:1000), anti-XPD (ABclonal: A19241, 1:1000), anti-XPB (ABclonal: A1714, 1:1000), anti-XPC (ABclonal: A8354, 1:1000), anti-ERCC1 (ABclonal: A4971, 1:1000), anti-RPA1 (ABclonal: A0990, 1:1000), anti-L-pan-Kla (PTM Biolab: PTM-1401RM, 1:1000) [3], anti-acetyl lysine (PTM Biolab: PTM-102, 1:1000) [19], anti-crotonyl lysine (PTM Biolab: PTM-502, 1:1000) [20], anti-succinyl lysine (PTM Biolab: PTM-419, 1:1000) [21], anti-LDHA (Cell Signaling Technology: no. 2012S, 1:1000), anti-LDHB (Millipore Sigma: ABC927, 1:1000), anti-KAT5 (Abmart: PA5312, 1:1000), anti-H3 (Proteintech, no. 17168-1-AP, 1:2000), and anti-XPA (Proteintech: 16462-1-AP, 1:1000).

### Plasmids and reagents

XPA WT and XPA KR mutants were subcloned into PLVX3 and PEGX-4T-2 vectors (Clontech). XPA KR mutants were established by a two-step mutation method. Briefly, first generate the K141/K145 double mutant, followed by introducing additional mutations at residues 217, 218, 221, and 222 to create the KR mutant. The acetyl-transferase plasmids CBP, P300, GCN5, PCAF, KAT5, and KAT8 were gifts from Dr Jun Huang. Sodium l-lactate (71718), LDHi (O2751), and puromycin (540222) were purchased from Sigma–Aldrich. MG149 (HY-15887) and MCT1/4i (HY-N4115) were purchased from MCE. Cy5-DNA_37_-F: 5′-phosphate-CCGAGTCATTCCTGCAGCGAGTCCATGGGAGTCAAAT, and Cy5-DNA_37_-R: 5′-Cy5-ATTTGACTCCCATGGACTCGCTGCAGGAATGACTCGG were purchased from Shanghai Generay Biotechnology. Cy5-DNA_37_ was prepared by annealing Cy5-DNA_37_-F and Cy5-DNA_37_-R in a reaction (1.25 μM top strand, 1 M bottom strand, 10 mM Tris–HCl, pH 8.0, and 100 mM KCl), incubated at 95°C for 5 min, and then cooled slowly to room temperature.

### Co-immunoprecipitation and western blot

For transient transfection and co-immunoprecipitation assays, constructs encoding GFP-Flag-tagged XPA or HA-tagged KAT5 plasmids were transiently co-transfected into HEK293T cells. The transfected cells were lysed with NETN buffer [20 mM Tris–HCl, pH 8.0, 100 mM NaCl, 1 mM ethylenediaminetetraacetic acid (EDTA), and 0.5% Nonidet *P*-40] containing 20 mM NaF and 1 g/ml phenylmethanesulfonyl fluoride on ice for 30 min. After removal of cell debris by centrifugation at 12 000 rpm for 10 min, the soluble fractions were collected and incubated with Flag beads for 2 h at 4°C. Beads were washed three times with NETN buffer, boiled in 1× sodium dodecyl sulfate (SDS) loading buffer for 10 min, and resolved on sodium dodecyl sulphate–polyacrylamide gel electrophoresis (SDS–PAGE). Membranes were blocked using 5% milk in TBST buffer and then probed with antibodies as indicated. Western blot experiments were performed in three independent biological replicates. The corresponding results are presented in the manuscript in the form of representative immunoblot images alongside quantitative statistical graphs.

### Mass spectrometry analysis

To identify XPA lactylation sites, HEK293T cells transiently expressing GFP-Flag-tagged XPA were treated with NALA for 24 h. Then harvested cells were lysed with NETN buffer containing 20 mM NaF and 1 g/ml phenylmethanesulfonyl fluoride on ice for 30 min. After removal of cell debris by centrifugation at 12 000 rpm for 10 min, the soluble fractions were collected and incubated with Flag beads for 2 h at 4°C. The pellet was then resolved on SDS–PAGE and stained with Coomassie blue staining. The band corresponding to GFP-Flag-tagged XPA was excised and sent for mass spectrometry (MS) analysis.

### *In vitro* acylation assay

The GST fusion XPA proteins and the GST proteins purified from *E. Coli* cells were incubated with HA-tagged KAT5 proteins, which were purified from HEK293T cells in reaction buffer [50 mM HEPES (pH 7.8), 30 mM KCl, 0.25 mM EDTA, 5.0 mM MgCl_2_, 5.0 mM sodium butyrate, 2.5 mM DTT) with increasing concentrations of lactyl-CoA or acetyl-CoA. Reactions were incubated at 30°C for 30 min. Next, 5×SDS loading buffer was added to the reaction and boiled for 10 min at 100°C. Samples were separated by SDS–PAGE and immunoblotted with indicated antibodies.

### Whole-cell extract and chromatin fractions extraction

First, cells were lysed with NETN buffer (20 mM Tris–HCl, pH 8.0, 100 mM NaCl, 1 mM EDTA, and 0.5% Nonidet *P*-40) containing 20 mM NaF and 1 g/ml phenylmethanesulfonyl fluoride on ice for 10 min. After the centrifugation (12 000 rpm for 10 min at 4°C), the supernatant was collected as the whole cell extract. The cell pellets were washed three times with 1×PBS and further lysed with cold EBC2 buffer [50 mM Tris–HCl (pH 7.5), 300 mM NaCl, 5 mM CaCl_2_, and 10 U microcal nuclease]. After sonication and centrifugation (12 000 rpm for 10 min at 4°C), the supernatants were collected as chromatin fractions.

### Electrophoretic mobility shift assay

The indicated XPA proteins were incubated with a 37 bp DNA substrate in reaction buffer (20 mM HEPES, pH 7.5, 150 mM NaCl, 5 mM DTT, 0.5 mg/ml BSA, and 5% glycerol) at 30°C for 30 min. After incubation, the reactions were mixed with loading buffer (20 mM Tris–HCl, pH 7.5, 40% glycerol, 2 mM EDTA, 0.2% orange G) and then loaded on 4% non-denaturing polyacrylamide gels (37.5:1 acrylamide: bis). Run were performed in 1×TAE buffer at constant voltage (90 V), as previously published [22].

### Proximity ligation assay

HeLa cells stably expressing Flag-tagged XPA WT or Flag-tagged XPA 6KR were treated with NALA or LDHi for 24 h. Then, cells were irradiated with UV through 5-micron filters and allowed to recover for 3 h. After being rinsed with 1×PBS, the cells underwent permeabilization with 0.5% Triton X-100 for 5 min, fixation with 3% formaldehyde for 10 min, and blocking using 3% BSA for 30 min at room temperature. After washing with 1× PBS, the cells were incubated with anti-Flag and anti-CPD antibody at 4°C overnight. The proximity ligation assay (PLA) was performed using the Duolink In Situ Red Starter Kit (Sigma–Aldrich) according to the manufacturer’s instructions. Images were acquired using a fluorescence microscope and analyzed accordingly. The displayed results represent the averages of three separate experiments and were presented as mean values ± SEM normalized to the WT group treated with UV irradiation. More than 200 cells were counted for each condition.

### Structural modeling and analysis

The structure of the XPA DNA-binding domain (residues M98-T239) was predicted using AlphaFold3. To ensure comprehensive conformational sampling, 500 independent models were generated using random seeds and executed on an NVIDIA RTX 3090 GPU. All selected models exhibited high prediction confidence (score > 0.8). The modeling accuracy was validated by structurally aligning the wild-type prediction with the reference X-ray crystal structure of XPA (PDB: 6J44). For targeted modification and mutational analyses, specific lysine residues (K141, K145, K217, K218, K221, and K222) were *in silico* lactylated (Chemical Component Dictionary ID: XRW) or substituted with arginine (K-to-R). Surface electrostatic potentials for all structural variants were computed and visualized using UCSF ChimeraX.

### Local UV irradiation

The HeLa cells on coverslips were washed and then covered with an Isopore polycarbonate membrane filter (pore size, 5 μm, Millipore) and subsequently exposed to UV irradiation (254 nm). Following the removal of the filter, the cells were cultivated for the indicated time. After being rinsed with 1× PBS, the cells underwent fixation with 4% formaldehyde for 10 min, permeabilization with 0.5% Triton X-100 for 5 min, and treatment with 2.5 M HCl for an additional 10 min to denature DNA. Thereafter, the cells were blocked using 5% BSA and incubated with primary antibodies against CPD. After washing three times with 1 ×PBS, the secondary antibody was incubated for another 30 min at room temperature. After washing three times with 1×PBS, cells were incubated with DAPI at room temperature for about 2 min. The cellular images were captured using a Nikon fluorescence microscope (Nikon). Statistical significance was determined with a two-tailed *t*-test. A *P*-value <0.05 was considered significant. * means *P* < .05, ** means *P* < .01.

### Dot-blot analysis

Dlot-blot analysis was performed essentially as described (Fan and Luo, 2008). Briefly, cells were harvested at indicated time points after UV irradiation. Genomic DNA was extracted using DNeasy kit (Vazyme) according to the protocol. For the control, DNA was harvested from unirradiated cells. DNA was denatured for quantification of thymine dimers as follows: DNA 50 ng, 0.4 M NaOH, 100 mM EDTA in final volume of 50 μl) was boiled for 10 min, followed by the addition of cold ammonium acetate to final concentrations of 2 M. Denatured was vacuum-transferred to nitrocellulose membrane (Beyotime) prewetted with 6×SSC buffer using Bio-dot microfitration apparatus (Bio-Rad). DNA was crosslinked to the membrane by incubation at 80°C for 2 h under vacuum. Quantification of thymine dimers was carried out using CPD antibody (MBL). Antibody binding was visualized by ECL method (ShareBio).

### Cell viability assay

Cells were seeded in 96-well plates (5000/well), allowed to attach overnight, then treated with indicated reagents (NALA 10 mM or 20 mM, LDHi 10 mM or 20 mM, MG149 25 mM) and increasing doses of UV irradiation or cisplatin, and then left to grow for another 48 h. Add 100 μl of medium containing 10% CCK8 [17]. After 2 h, measure the absorbance at 450 nm using a spectrophotometric plate reader (Thermo). Each assay was performed in three independent biological replicates, and representative images are displayed. For statistical analysis of significance between two groups, a Student’s t-test was performed. For statistical analysis of significance between multiple groups, one way or two-way analysis of variance (ANOVA) was used. The value of *P* < .05 was significant. * means *P* < .05, ** means *P* < .01. ns means no significant change.

## Results

### Induced lactylation promotes NER and UV resistance in cancer cells

As a newly identified type of post-translational modification, lactylation has been reported to play a critical role in regulating immunity [23], cell proliferation [24], HR repair [7], and other processes. However, whether the protein lactylation is involved in UV-mediated DNA damage response remains unknown. We manipulated protein lactylation in cells via treatment with NALA (sodium l-lactate) or a lactate dehydrogenase inhibitor (LDHi), and further investigated the role of lactylation in UV-induced DNA damage repair by comparing the repair rates of CPDs. As shown in Fig. 1A and B, NALA treatment increased the CPD repair rate, whereas LDHi delayed the CPD repair rate. Furthermore, we observed that NALA treatment accelerated the kinetics of CPD repair relative to the untreated group, whereas LDHi treatment slowed CPD repair kinetics (Supplementary Figs S1A and B, and S7A and B). In addition, knockdown of lactate dehydrogenase A/B (LDHA/B), a manipulation previously reported to inhibit lactate production and thereby reduce protein lactylation [3, 25], was found to impede the removal of CPDs (Fig. 1C–F and Supplementary Fig. S6A and B). Moreover, NALA treatment both promoted protein lactylation and increased the resistance of cells to UV irradiation in a dose-dependent manner (Fig. 1G–I and Supplementary Figs S1C–E, S6C and D, and S7C and D). Conversely, LDHi treatment both decreased protein lactylation and significantly rendered tumor cells hypersensitive to UV irradiation in a dose-dependent manner (Fig. 1J–L and Supplementary Figs S1F–H, S6E and F, and S7E and F). Additionally, LDHA/B depletion showed similar phenomenon as LDHi treatment (Fig. 1M–O and Supplementary Figs S1I–K, S6G and H, and S7G and H). Taken together, these findings suggest that lactate-mediated protein lactylation may play a pivotal role in regulating UV-induced damage repaired by NER.

**Figure 1.**
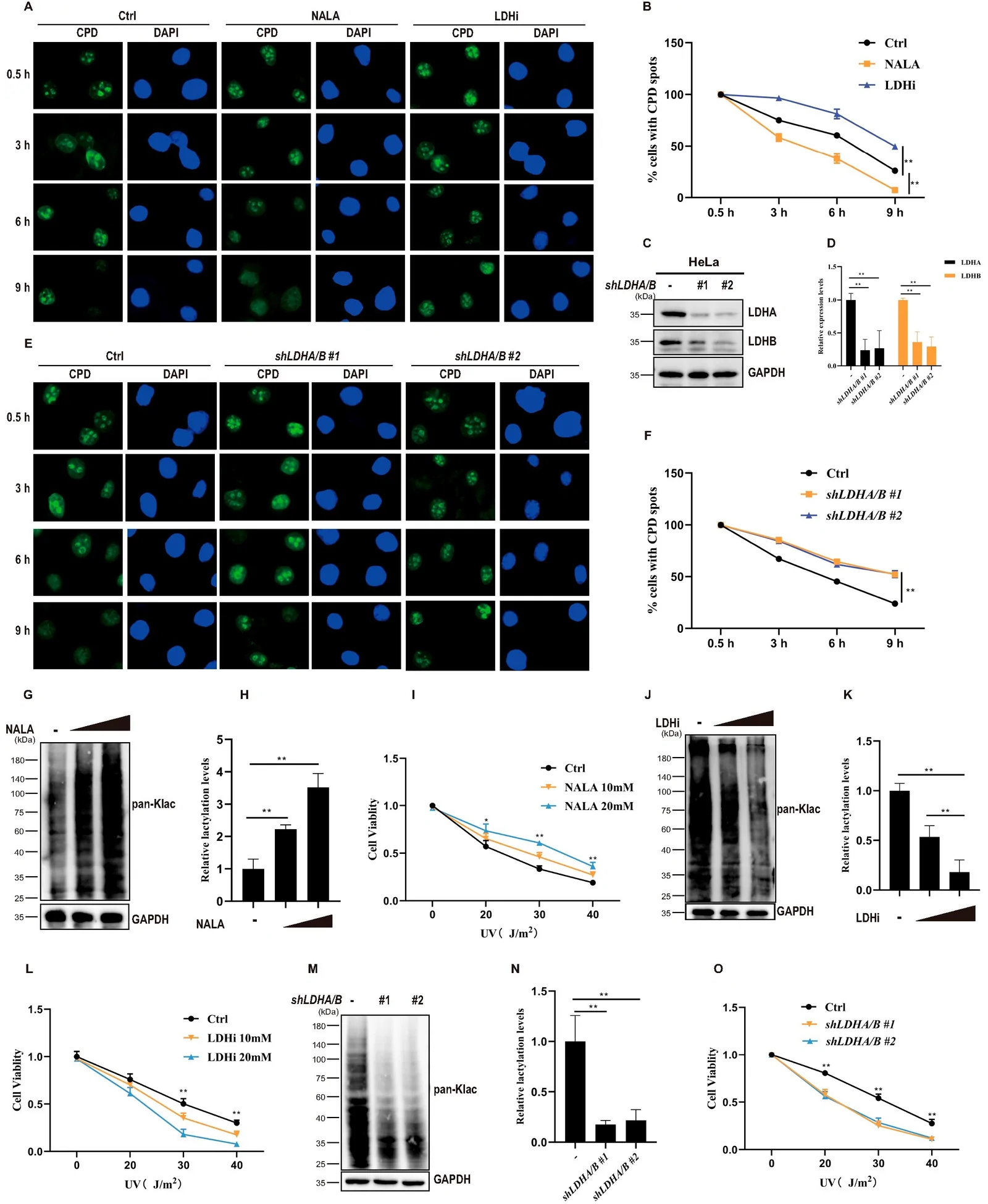
Induced lactylation promotes NER and UV resistance in cancer cells. (**A**) HeLa cells were treated with NALA or LDHi for 24 h. Then cells were irradiated with UV through 5-micron filters and allowed to recover for indicated times before being stained with CPD antibody. (**B**) The displayed results represent the averages of three separate experiments and were presented as mean values ± SEM. More than 200 cells were counted for each condition. (**C**) Western blot with indicated antibodies in HeLa cells stably LDHA/B knockdown. (**D**) Quantification of the indicated protein levels from three independent experiments related to panel (C). (**E**) Stably depleted of LDHA/B or control cells were irradiated with UV through 5-micron filters and allowed to recover for indicated times before being stained with CPD antibody. (**F**) The displayed results represent the averages of three separate experiments and were presented as mean values ± SEM. More than 200 cells were counted for each condition. (**G**) HeLa cells were exposed to a gradient concentration of NALA or vehicle for 24 h, followed by lysis. Subsequently, immunoblotting was carried out using the indicated antibodies. (**H**) Quantification of the lactylation levels from three independent experiments related to panel (G). (**I**) HeLa cells were treated with increasing doses of NALA and UV irradiation, followed by 48-h recovery. CCK8 assay was performed as detailed in the Methods. (**J**) HeLa cells were exposed to a gradient concentration of LDHi or vehicle for 24 h, followed by lysis. Subsequently, immunoblotting was carried out using the indicated antibodies. (**K**) Quantification of the lactylation levels from three independent experiments related to panel (J). (**L**) HeLa cells were treated with increasing doses of LDHi and UV irradiation, followed by 48-h recovery. CCK8 assay was performed as detailed in the “Materials and methods” section. (**M**) HeLa cells stably depleted of LDHA/B or control cells from panel (C) were lysed and then the immunoblotting was performed with indicated antibodies. (**N**) Quantification of the lactylation levels from three independent experiments related to panel (M). (**O**) Cells from panel (M) were utilized to perform CCK8 as indicated.

### XPA is lactylated by KAT5

To investigate the underlying mechanism by which protein lactylation regulates NER, we screened several crucial factors implicated in the NER process and determined that XPA presented the highest level of lactylation (Fig. 2A and B, and Supplementary Fig. S8A and B). In addition, we found that NALA treatment notably enhanced the lactylation of XPA, whereas LDHi treatment markedly decreased XPA lactylation without affecting other acylation of XPA, such as acetylation [19], crotonylation [20], and succinylation [26] (Fig. 2C and D, and Supplementary Figs S2A and B, S8C and D, and S9A and B). Consistently, we observed that endogenous XPA also undergoes lactylation, as demonstrated in Fig. 2C (Supplementary Figs S2C and D, and S9C and D). LDHA/B knockdown significantly reduced the lactylation of XPA, and this reduction was partially reversed by the addition of NALA (Fig. 2E and F, and Supplementary Fig. S8E and F). Furthermore, the results from the biotin pull-down assay ruled out the possibility of a direct interaction between XPA and lactic acid (Supplementary Figs S2E and S9E and F). MCT1/4 inhibitor treatment, which was reported to elevate the lactate level in cells [7], also remarkedly increased lactylation of XPA (Supplementary Figs S2F and G, and S9G and H). Furthermore, the lactylation of XPA significantly increased after UV irradiation (Fig. 2G and H, and Supplementary Figs S2H and I, S8G and H, and S9I and J), as well as after treatment with cisplatin or mitomycin C agents that induce interstrand crosslink and DNA adducts (Supplementary Figs S2J and K, and S9K and L). These findings implied that XPA could be lactylated and that this lactylation was induced by UV irradiation. Given that lactylation is a type of lysine acylation modification, common acyltransferases [5, 7, 27–30] may also serve as “writer(s)” for lactylation. Considering that p300, CBP, KAT5, KAT8, GCN5, and PCAF are well-known and commonly studied acyltransferases, and prior literature has reported their potential functions as lactyltransferases, we prioritized these six enzymes for initial screening. After screening, the results revealed that KAT5 was a potential “writer” for the lactylation of XPA (Fig. 2I and J, and Supplementary Fig. S8I and J). To investigate the role of KAT5 in XPA lactylation, cells were treated with the KAT5 inhibitor MG149, and the results showed that MG149 treatment notably inhibited XPA lactylation (Fig. 2K and L, and Supplementary Figs S2L and M, S8K and L, and S9M and N). In addition, KAT5 depletion decreased XPA lactylation (Fig. 2M and N, and Supplementary Fig. S8M and N). We further demonstrated an endogenous interaction between KAT5 and XPA (Fig. 2O and P, and Supplementary Figs S2N and O, S8O–R, and S9O–R). To confirm that KAT5 is the lactyltransferase for XPA, we conducted an *in vitro* lactylation assay. As shown in Fig. 2Q and R, and Supplementary Figs S2P and S8S and T, KAT5 directly and specifically catalyzes the lactylation, not acetylation, of XPA in a lactyl-CoA dose-dependent manner. Furthermore, we observed that zinc ions supplementation, which has been reported to potentiate the catalytic activity of KAT5 [31–33], leaded to a marked increase in XPA lactylation, while XPA acetylation remained unaltered (Supplementary Figs S2Q and S9S). Additionally, as a known acetylation substrate of KAT5 [34], p53 exhibits strongly potentiated KAT5-mediated acetylation upon zinc treatment in *in vitro* assay (Supplementary Figs S2R and S9T). These findings suggest that KAT5 specifically catalyzed the lactylation but not acetylation of XPA. Taken together, we concluded that KAT5 is the major lactyltransferase for XPA.

**Figure 2.**
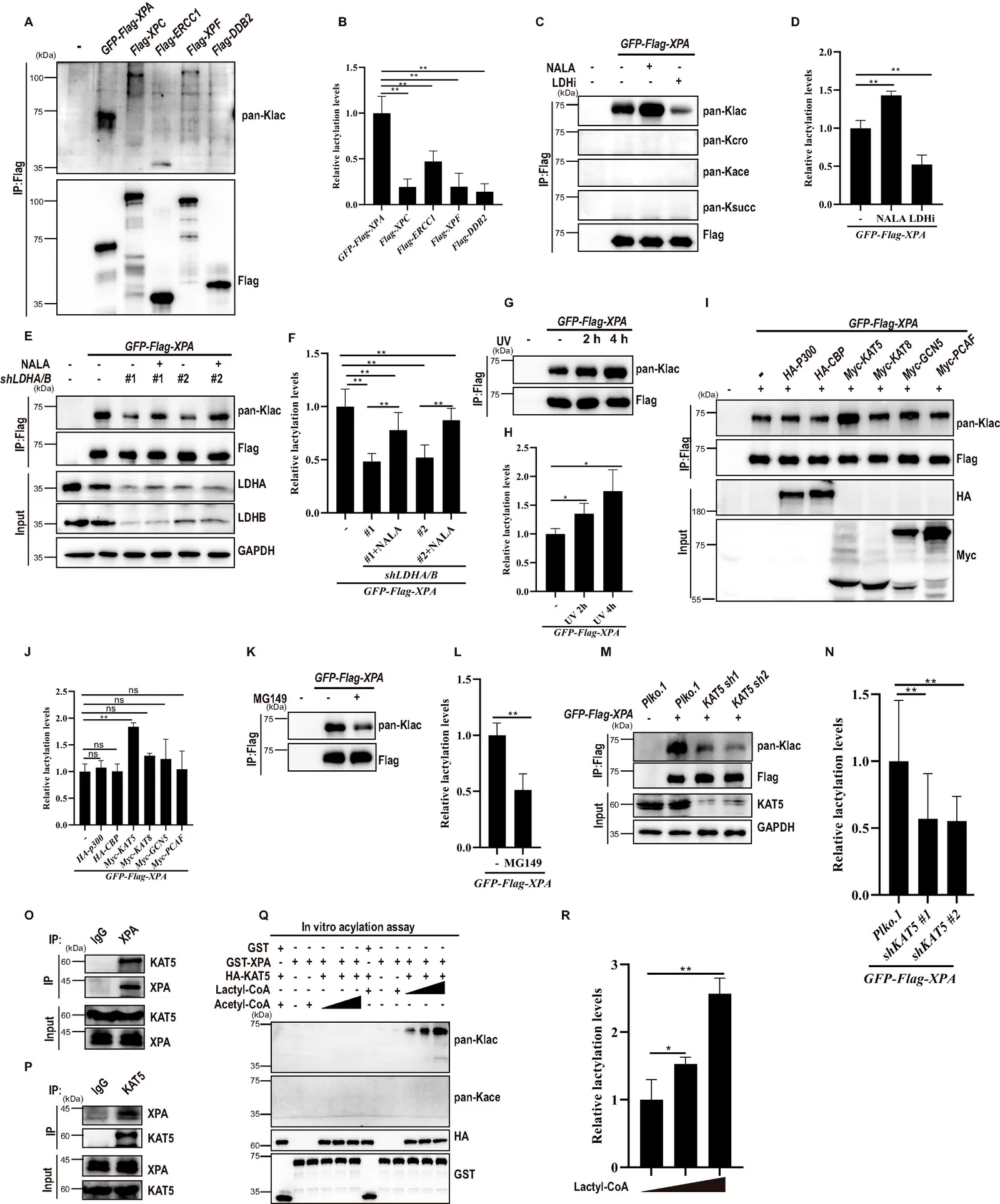
XPA is lactylated by KAT5. (**A**) HEK293T cells were transfected with indicated plasmids, then cells were lysed and immunoprecipitated with anti-Flag agarose beads after 48 h. The precipitates were analyzed by immunoblotting using the indicated antibodies. (**B**) Quantification of the lactylation levels from three independent experiments related to panel (A). (**C**) HEK293T cells were transfected with GFP-Flag-tagged XPA and treated with either LDHi or NALA for 24 h before lysis. Cell lysates were immunoprecipitated with anti-Flag agarose beads, and western blot analysis was performed with indicated antibodies. (**D**) Quantification of the lactylation levels from three independent experiments related to panel (C). (**E**) HEK293T cells stably depleted of LDHA/B or control cells were transfected with GFP-Flag-tagged XPA and treated with NALA for 24 h before lysis. Cell lysates were immunoprecipitated with anti-Flag agarose beads, and western blot analysis was performed with indicated antibodies. (**F**) Quantification of the lactylation levels from three independent experiments related to panel (E). (**G**) HEK293T cells were transfected with GFP-Flag-tagged XPA and treated with UV (60 J/m^2^) irradiation with indicated time before lysis. Cell lysates were immunoprecipitated with anti-Flag agarose beads, and western blot analysis was performed with indicated antibodies. (**H**) Quantification of the lactylation levels from three independent experiments related to panel (G). (**I**) HEK293T cells were transiently transfected with GFP-Flag-tagged XPA together with plasmids encoding Myc- or HA-tagged acetyltransferases as indicated. Cell lysates were immunoprecipitated with anti-Flag agarose beads, and western blot analysis was performed with indicated antibodies. (**J**) Quantification of the lactylation levels from three independent experiments related to panel (I). (**K**) HEK293T cells were transfected with GFP-Flag-tagged XPA and treated with the KAT5 inhibitor MG149 (100 μM) for 2 h before lysis. Cell lysates were immunoprecipitated with anti-Flag agarose beads, and western blot analysis was performed with indicated antibodies. (**L**) Quantification of the lactylation levels from three independent experiments related to panel (K). (**M**) HEK293T cells depleted of KAT5 or control cells were transfected with GFP-Flag-tagged XPA. Cell lysates were immunoprecipitated with anti-Flag agarose beads, and western blot analysis was performed with indicated antibodies. (**N**) Quantification of the lactylation levels from three independent experiments related to panel (M). (**O**) HeLa cells were lysed and then purified with Protein A and G agarose beads pre-coupled with rabbit IgG (control) or anti-XPA antibody. Whole-cell lysis and the immunoprecipitates were then blotted with the indicated antibodies. (**P**) HeLa cells were lysed and then purified with Protein A and G agarose beads pre-coupled with rabbit IgG (control) or anti-KAT5 antibody. Whole-cell lysis and the immunoprecipitates were then blotted with the indicated antibodies. (**Q**) GST (control) and GST-XPA proteins purified from *E. coli* were incubated with HA-tagged KAT5 purified from HEK293T cells together with increasing dose of lactyl-CoA or acetyl-CoA in *in vitro* acylation buffer at 30°C for 30 min. The reaction mixture was analyzed by western blot with indicated antibodies. (**R**) Quantification of the lactylation levels from three independent experiments related to panel (Q).

### XPA is lactylated at lysines located in the DNA-binding domain

XPA is a crucial NER protein that consists of an N-terminal domain, a central globular core domain, a DNA-binding domain, and a C-terminal domain (Fig. 3A). To identify the lactylation sites of XPA, we purified Flag-tagged XPA through immunoprecipitation and then performed MS analysis. As shown in Fig. 3A and Supplementary Fig S3A–S3E, six possible lactylation sites (K141, K145, K217, K218, K221, and K222) located in the DNA-binding domain of XPA were identified by MS. We then investigated the contributions of these six sites to XPA lactylation. First, we discovered that the substitution of lysine (K) with arginine (R) as a single mutation at these six sites, each on its own, caused a relatively mild or small reduction in the lactylation of XPA (Fig. 3B and C, and Supplementary Fig. S10A and B). Hence, these six sites might be the major sites for XPA lactylation. To test this, we constructed combined K-to-R mutants at the six sites (6KR). As shown in Fig. 3D and E, and Supplementary Fig S10C and D, compared with XPA WT, for the XPA 6KR mutant, lactylation was significantly reduced, and the NALA-induced increase in lactylation was prevented. KAT5 overexpression markedly increased XPA WT lactylation but not XPA 6KR mutant lactylation (Fig. 3F and G, and Supplementary Fig. S10E and F). Conversely, KAT5 knockdown significantly reduced XPA WT lactylation but not XPA 6KR mutant lactylation (Fig. 3H and I, and Supplementary Fig. S10G and H). Furthermore, the lactylation was enhanced in response to UV irradiation in XPA WT but not XPA 6KR mutant (Fig. 3J and K, and Supplementary Fig. S10I and J). To further validate these lactylation sites, an *in vitro* lactylation assay was conducted using GST-XPA and the GST-XPA 6KR mutant proteins. KAT5 catalyzed the lactylation of GST-XPA WT but not the lactylation of the GST-XPA 6KR mutant (Fig. 3L and M, and Supplementary Figs S3F and S10K and L). Taken together, these results demonstrated that the six lysine residues, as K141, K145, K217, K218, K221, and K222, are the major sites for XPA lactylation in response to UV irradiation.

**Figure 3.**
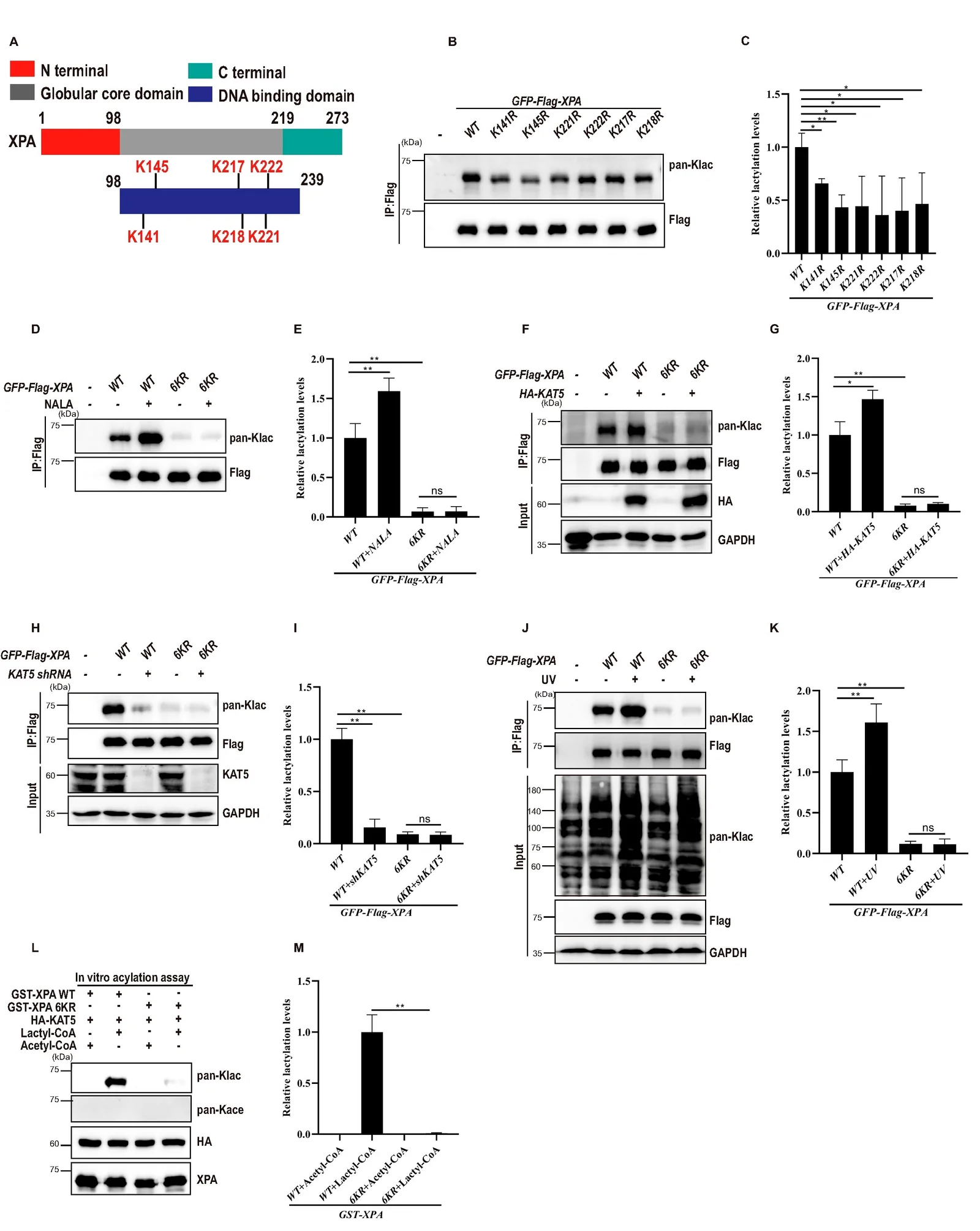
XPA is lactylated at six sites. (**A**) Schematic representation of XPA key domains and possible lactylation sites which were identified by MS analysis. (**B**) HEK293T cells were transfected with GFP-Flag-tagged XPA WT and indicated XPA mutants. Cell lysates were immunoprecipitated with anti-Flag agarose beads, and western blot analysis was performed with indicated antibodies. (**C**) Quantification of the lactylation levels from three independent experiments related to panel (B). (**D**) HEK293T cells were transfected with GFP-Flag-tagged XPA WT and XPA 6KR mutants. After 24 h transfection, cells were treated with NALA for 24 h. Cell lysates were immunoprecipitated with anti-Flag agarose beads, and western blot analysis was performed with indicated antibodies. (**E**) Quantification of the lactylation levels from three independent experiments related to panel (D). (**F**) HEK293T cells were transfected with GFP-Flag-tagged XPA WT or XPA 6KR together with HA-tagged KAT5, and then cells were lysed and immunoprecipitated with anti-Flag agarose beads after 48 h. The precipitates were analyzed by immunoblotting using the indicated antibodies. (**G**) Quantification of the lactylation levels from three independent experiments related to panel (F). (**H**) HEK293T cells depleted of KAT5 were transfected with GFP-Flag-tagged XPA WT or XPA 6KR. After transfection for 48 h, cells were lysed and immunoprecipitated with anti-Flag agarose beads. The precipitates were analyzed by immunoblotting using the indicated antibodies. (**I**) Quantification of the lactylation levels from three independent experiments related to panel (H). (**J**) HEK293T cells were transfected with GFP-Flag-tagged XPA WT or XPA 6KR mutants and treated with UV (60 J/m^2^) irradiation 4 h before lysis. Cell lysates were immunoprecipitated with anti-Flag agarose beads, and western blot analysis was performed with indicated antibodies. (**K**) Quantification of the lactylation levels from immunoprecipitates of three independent experiments related to panel (J). (**L**) GST-XPA WT or GST-XPA 6KR proteins purified from *E. coli* were incubated with HA-tagged KAT5 purified from HEK293T cells together with lactyl-CoA or acetyl-CoA in *in vitro* acylation buffer at 30°C for 30 min. The reaction mixture was analyzed by western blot with indicated antibodies. (**M**) Quantification of the lactylation levels from three independent experiments related to panel (L).

### Lactylation enhances the ability of XPA to bind DNA

We subsequently investigated whether lactylation regulates the function of XPA. Previous studies have reported that post-translational modifications can regulate protein stability and expression. To investigate whether XPA lactylation affects its stability, we inhibited protein synthesis with cycloheximide (CHX) and measured the half-life of XPA. Results showed that the half-life of XPA remained unchanged in cells treated with either NALA or LDHi (Fig. 4A and B, and Supplementary Fig. S11A and B). The NER process involves interactions among multiple repair proteins, with XPA serving as a pivotal scaffolding protein. During NER, XPA binds to multiple proteins, thereby influencing the recruitment, dissociation, and activation of other repair proteins. We first found that neither NALA nor LDHi affected the interactions between XPA and XPC, XPB, XPD, RPA2, RPA1, or ERCC1 (Fig. 4C and D, and Supplementary Fig. S11C and D). Considering that XPA contains a DNA-binding domain and that all lactylation sites are located within this DNA-binding domain, we wondered whether NALA and LDHi regulate the recruitment of XPA to chromatin. Interestingly, we found that under UV irradiation, NALA treatment promoted XPA chromatin loading, whereas LDHi treatment decreased its chromatin recruitment (Fig. 4E–H and Supplementary Fig. S11E–1H). Moreover, inhibition of KAT5 by MG149, KAT5 knockdown, or LDHA/B depletion dramatically reduced XPA chromatin loading under UV irradiation (Fig. 4I–L and Supplementary Figs S4A–D, S11I–L, and S12A–D). However, KAT5 overexpression increased XPA chromatin recruitment under UV irradiation (Supplementary Figs S4E and F, and S12E and F). As shown in Fig. 4M and N, electrophoretic mobility shift assay (EMSA) results suggested that XPA lactylation strengthens the binding capacity of XPA to DNA, relative to the XPA 6KR mutant. To further explore the function of XPA lactylation in chromatin loading, we constructed cancer cells that stably expressed XPA WT or the XPA 6KR mutant in which endogenous XPA was depleted (Supplementary Figs S4G and H, and S12G and H). Compared with that of XPA WT, the recruitment of XPA 6KR to chromatin was significantly reduced under UV irradiation. Furthermore, NALA treatment markedly enhanced the binding of XPA WT to chromatin, and LDHi treatment inhibited this binding; in contrast, XPA 6KR remained unaffected (Fig. 4O and P, and Supplementary Figs S4I and J, S11M and N, and S12I and J). The KAT5 inhibitor MG149 reversed the effect of NALA on the recruitment of XPA WT to chromatin but had no significant effect on XPA 6KR (Fig. 4Q and R, and Supplementary Figs S4K and L, S11O and P, and S12K and L). Furthermore, overexpression of KAT5 enhances the recruitment of XPA to chromatin in the XPA WT group, but exhibits no notable effect on the XPA 6KR mutant (Supplementary Figs S4M and N, and S12M and N). As shown in Supplementary Fig. S4O and P, under UV irradiation, compared with XPA 6KR mutant, NALA markedly elevated PLA signals in the XPA WT cells yet exerted no effect on XPA 6KR cells. Conversely, LDHi significantly reduced PLA signals in the XPA WT cells without impacting XPA 6KR cells. In addition, structural analysis revealed that lactylation of XPA has no significant effect on the stability or conformation of its DNA-binding domain (Supplementary Fig. S4Q), and that these lactylation sites are located at or near the DNA-binding interface (Supplementary Fig. S4R), which may contribute to enhancing the interaction between XPA and DNA. Compared with XPA-WT, the XPA-6KR mutation did not significantly alter the global structural conformation or the surface electrostatic potential of XPA (Supplementary Fig. S4S and T). To further explore how XPA lactylation regulates NER, given that XPA serves as a scaffold for the recruitment of downstream NER factors [35, 36], we examined the chromatin recruitment of two important downstream NER effector proteins (XPF and XPG, the XPA–dependent downstream nucleases, are essential components of pre-incision complex [35, 37, 38]) in XPA-WT cells and XPA-6KR cells under UV irradiation. Our results demonstrate that the recruitment of XPF and XPG to chromatin is markedly attenuated in XPA–6KR cells compared with XPA–WT cells (Supplementary Figs S4U and S12O). These findings demonstrated that the lactylation of XPA can promote the recruitment of XPA to chromatin under UV irradiation, thereby facilitating the recruitment of downstream factors, driving pre-incision complex formation, and suggesting its potential role in enhancing the DNA damage repair process.

**Figure 4.**
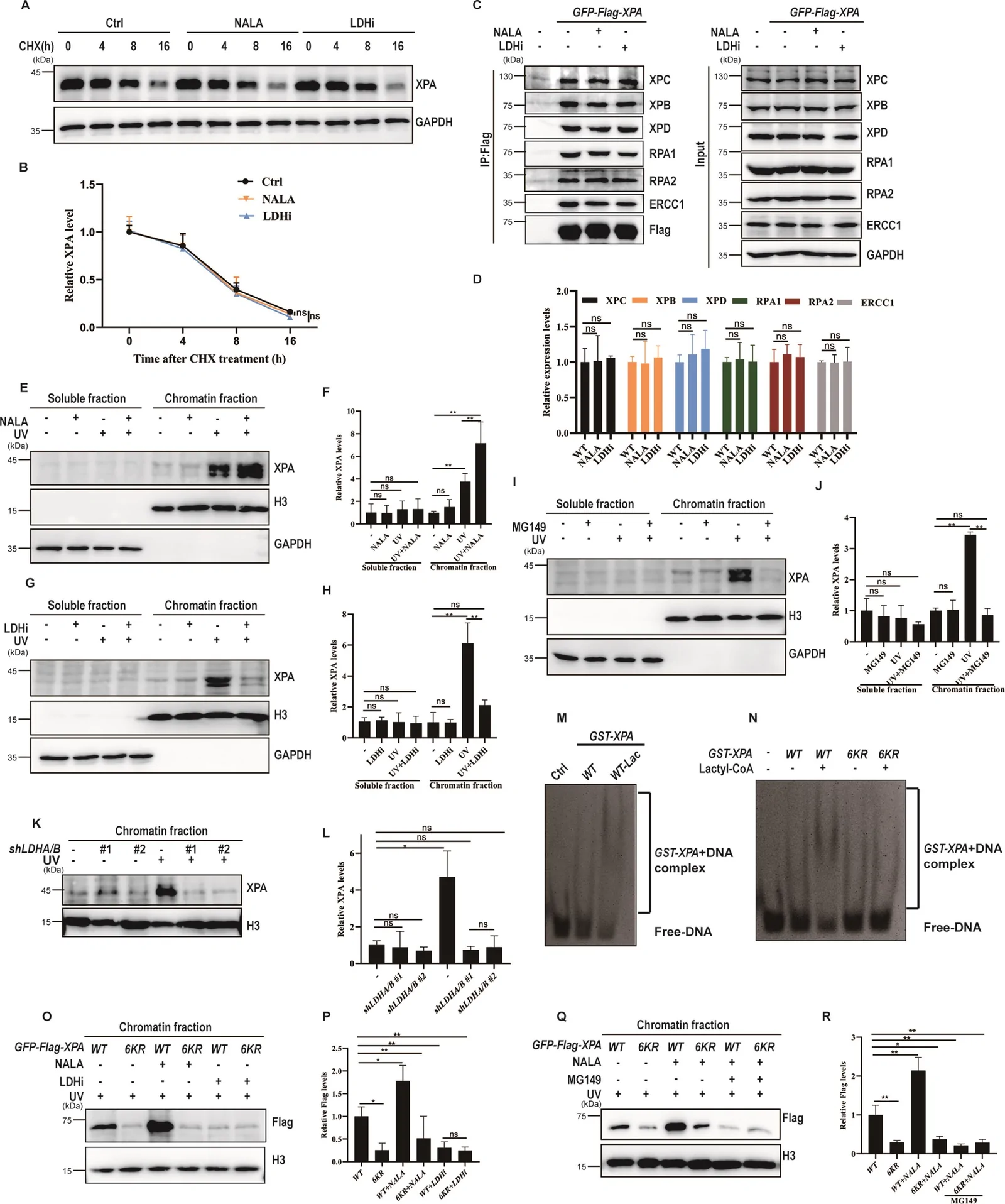
Lactylation enhances the ability of XPA to bind DNA. (**A**) HeLa cells pre-treated with NALA or LDHi were then exposed to CHX (0.1 mg/ml) and harvested at specified time points, and western blot analysis was performed with indicated antibodies. (**B**) Quantification of the XPA protein levels from three independent experiments related to panel (A). (**C**) HEK293T cells were transfected with GFP-Flag-tagged XPA. After treatment with NALA or LDHi for 24 h. Cell lysates were immunoprecipitated with anti-Flag agarose beads, and western blot analysis was performed with indicated antibodies. (**D**) Quantification of the indicated protein levels from three independent experiments related to panel (C). (**E**) After treatment with NALA for 24 h, HeLa cells were irradiated with UV (60 J/m^2^) and then allowed to recover for 4 h. Cell lysates were divided into soluble fraction and chromatin fraction. These two components were blotted with indicated antibodies. (**F**) Quantification of the XPA levels from three independent experiments related to panel (E). (**G**) After treatment with LDHi for 24 h, HeLa cells were irradiated with UV (60 J/m^2^) and then allowed to recover for 4 h. Cell lysates were divided into soluble fraction and chromatin fraction. These two components were blotted with indicated antibodies. (**H**) Quantification of the XPA levels from three independent experiments related to panel (G). (**I**) After treatment with MG149 for 1 h, HeLa cells were irradiated with UV (60 J/m^2^) and then allowed to recover for 4 h. Cell lysates were divided into soluble fraction and chromatin fraction. These two components were blotted with indicated antibodies. (**J**) Quantification of the XPA levels from three independent experiments related to panel (I). (**K**) HeLa cells stably depleted of LDHA/B were irradiated with UV (60 J/m^2^) and then allowed to recover for 4 h. Cell lysates were divided into soluble fraction and chromatin fraction. These two components were blotted with indicated antibodies. (**L**) Quantification of the XPA levels from three independent experiments related to panel (K). (**M**) Representative EMSA gel showing unlactylated GST-XPA or lactylated GST-XPA binding to a 37 bp DNA substrate. (**N**) Representative EMSA gel showing lactylated GST-XPA WT or GST-XPA 6KR binding to a 37 bp DNA substrate as indicated. (**O**) Stably expressing GFP-Flag-tagged XPA WT or XPA 6KR HeLa cells were treated with the indicated drug and UV irradiation. Cell lysates were divided into soluble fraction and chromatin fraction. The chromatin fraction was blotted with indicated antibodies. (**P**) Quantification of the GFP-Flag-XPA levels from three independent experiments related to panel (O). (**Q**) Stably expressing GFP-Flag-tagged XPA WT or XPA 6KR HeLa cells were treated with the indicated drug and UV irradiation. Cell lysates were divided into soluble fraction and chromatin fraction. The chromatin fraction was blotted with indicated antibodies. (**R**) Quantification of the GFP-Flag-XPA levels from three independent experiments related to panel (Q).

### XPA lactylation promotes NER and cell survival under UV-induced damage

To investigate whether XPA lactylation is required for NER, we generated derivatives of HeLa cell lines that stably expressed XPA WT or XPA 6KR (Fig. 5A and B, and Supplementary Fig. S13A and ) and analyzed the CPD removal rate in XPA WT cells and XPA 6KR cells. As expected, compared with XPA WT cells, XPA 6KR cells displayed a much slower CPD removal rate (Fig. 5C and D). In XPA WT cells, NALA promoted the clearance of CPDs, while LDHi inhibited the clearance of CPDs (Fig. 5C and D). However, in XPA 6KR cells, there were no observed effects of NALA and LDHi. Compared with XPA WT cells, XPA 6KR cells exhibited slower CPDs repair kinetics. Moreover, NALA treatment promoted CPDs repair kinetics, but had no significant effect on XPA 6KR cells. Conversely, LDHi treatment delayed CPDs repair kinetics in XPA WT cells but had no significant effect on XPA 6KR cells (Supplementary Figs S5A and B, and S14A and B). Additionally, MG149 inhibited the clearance of CPDs in XPA WT cells, without significant difference in XPA 6KR cells (Fig. 5E and F). Given that the overactivation of DNA damage repair in cells can lead to resistance to DNA damage [7, 39], we investigated whether induced XPA lactylation promotes cell survival in response to UV-induced DNA damage. As shown in Fig. 5G and H, NALA treatment led to UV resistance in tumor cells; however, the administration of MG149 reversed this effect, enhancing UV sensitivity. Compared with XPA WT cells, XPA 6KR cells exhibited greater UV sensitivity (Fig. 5I–P and Supplementary Figs S5C–H and S14C–H). Moreover, NALA treatment promoted XPA WT cell resistance to UV irradiation but had no significant effect on XPA 6KR cells (Fig. 5I–L). Conversely, LDHi treatment enhanced the UV sensitivity of XPA WT cells but had no significant effect on XPA 6KR cells (Fig. 5M–P). MG149 treatment also sensitized cancer cells expressing XPA WT to UV irradiation, but not those expressing XPA 6KR (Supplementary Fig. S5I–L). Given that cisplatin-induced DNA adducts can also be cleared by NER, we examined whether XPA lactylation also affects tumor cell survival under cisplatin treatment. The results show the XPA 6KR cells were more sensitive to cisplatin than the XPA WT cells (Supplementary Fig. S5M–X). NALA treatment resulted in strong cisplatin resistance in cancer cells expressing XPA WT, but not those expressing XPA 6KR (Supplementary Fig. S5M–P). However, LDHi or MG149 treatment sensitized cancer cells expressing XPA WT to cisplatin, but not those expressing XPA 6KR (Supplementary Fig. S5Q–T). MG149 treatment also sensitized cancer cells expressing XPA WT to cisplatin, but not those expressing XPA 6KR (Supplementary Fig. S5U–X). Taken together, XPA lactylation is crucial for NER and enhances cell survival under UV irradiation, thereby protecting genomic stability and potentially promoting tumor tolerance to radiotherapy and chemotherapy.

**Figure 5.**
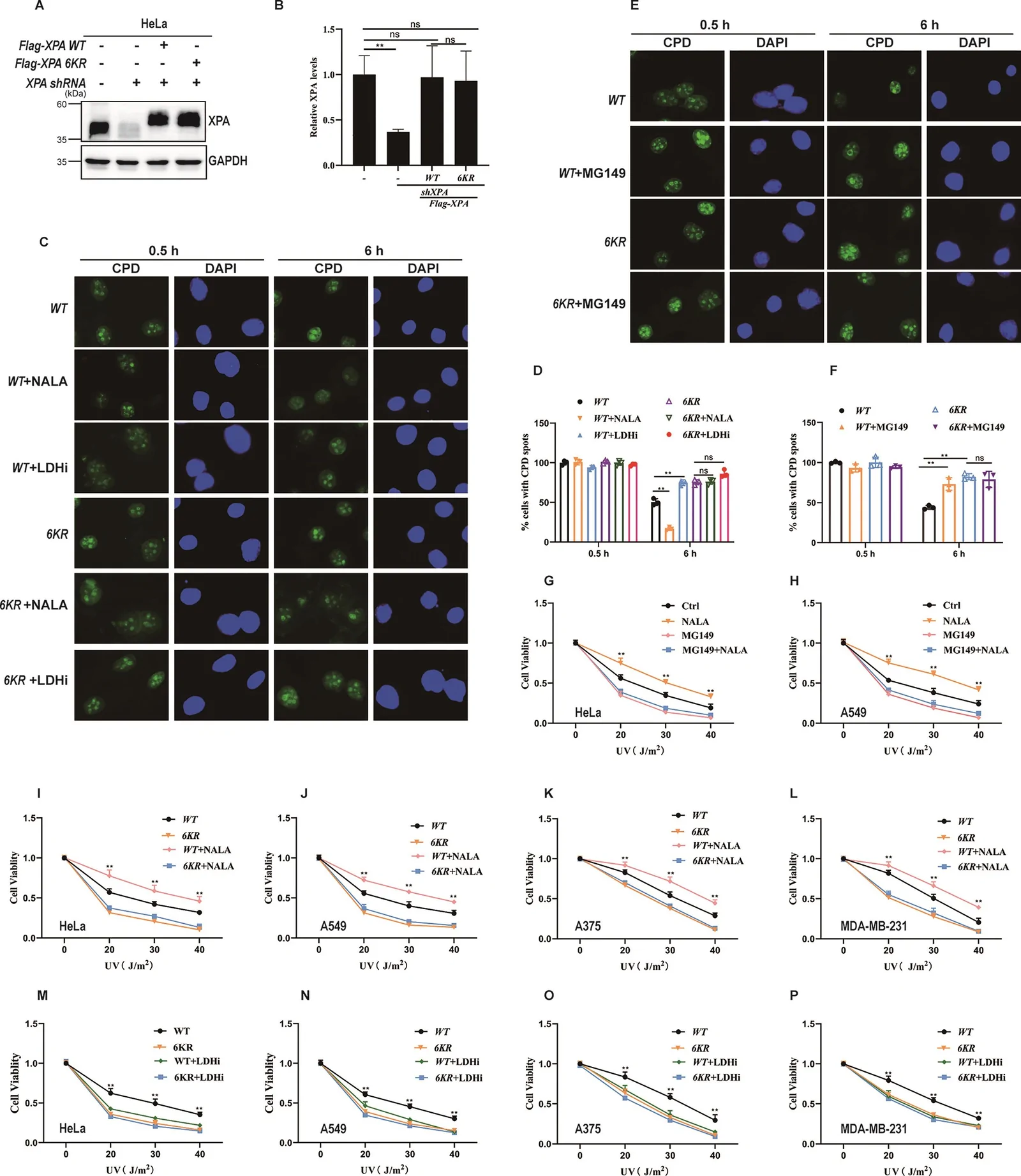
XPA lactylation promotes NER and cell survival under UV-induced damage. (**A**) Western blot with indicated antibodies in HeLa cells stably expressing Flag-tagged XPA WT or Flag-tagged XPA 6KR. (**B**) Quantification of the XPA levels from three independent experiments related to panel (A). (**C**) HeLa cells stably expressing XPA WT or XPA 6KR were treated with NALA or LDHi for 24 h. Then, cells were irradiated with UV through 5-micron filters and allowed to recover for the indicated times before being stained with antibodies for CPD. (**D**) The displayed results represent the averages of three separate experiments and were presented as mean values ± SEM. More than 200 cells were counted for each condition. (**E**) HeLa cells stably expressed XPA WT or XPA 6KR were treated with MG149 for 24 h. Then cells were irradiated with UV through 5-micron filters and allowed to recover for the indicated times before being stained with antibodies for CPD. (**F**) The displayed results represent the averages of three separate experiments and were presented as mean values ± SEM. More than 200 cells were counted for each condition. (**G**) HeLa cells were used for CCK8 analysis as indicated. (**H**) A549 cells were used for CCK8 analysis as indicated. Cell viability was assessed in XPA-WT or 6KR HeLa (**I**), A549 (**J**), A375 (**K**), and MDA-MB-231 (**L**) cells treated with NALA under UV irradiation. Cell viability was assessed in XPA-WT or 6KR HeLa (**M**), A549 (**N**), A375 (**O**), and MDA-MB-231 (**P**) cells treated LDHi under UV irradiation.

## Discussion

The Warburg effect, a hallmark of metabolic reprogramming in tumor cells, generates substantial amounts of lactate, which contributes to immune evasion [40], proliferation [41], metastasis [42], and resistance to radiotherapy and chemotherapy [39]; however, the specific mechanisms are still not fully elucidated. Lactylation, a newly discovered post-translational modification of histone lysine residues that can be induced by lactate [3], offers a potential mechanism for these effects.

Defects in NER lead to the accumulation of lesions throughout the genome, which can be bypassed by translesion DNA polymerases. Although bypassing these lesions can promote cell survival, the probability of gene mutations increases, facilitating tumorigenesis. Subtypes of xeroderma pigmentosum syndrome confer high susceptibility to cancer, with a 1000-fold increased risk of skin tumors and a significant increase in the risk of other types of tumors [43]. The regulation of XPA transcription, stability, and chromatin loading is vital for NER. XPA is a downstream target gene of HIF-1, and the binding of HIF-1 to the promoter region of XPA can promote the transcription of XPA; thus, the inhibition of HIF-1 can increase the sensitivity of ovarian cancer to cisplatin [44, 45]. In addition, upon UV irradiation, activated ATR phosphorylates XPA at S196, thereby enhancing the interaction between XPA and RPA1 and promoting resistance to UV in tumor cells [46]. Conversely, WIP1 can dephosphorylate XPA at S196, reducing NER activity and enhancing UV-induced apoptosis [47]. Furthermore, XPA can be ubiquitinated by HERC2, a process that can be inhibited by the ATR-mediated phosphorylation of XPA, leading to XPA degradation via the proteasome pathway, which inhibits NER [46, 48]. However, it is unclear whether tumor metabolism, especially lactate metabolism, can regulate the function of XPA and, in turn, affect NER repair.

Herein, we discovered that the key protein XPA was lactylated by the acetyltransferase KAT5 without affecting the acetylation, succinylation, or crotonylation of XPA. Additionally, the lactylation of XPA significantly increased in response to UV damage. Although KAT5 plays a critical role in NER through acetylation of XPF [49], we have found that it also regulates NER via lactylation of XPA, further expanding its regulatory mechanisms in NER. Besides NBS1 [39] and VPS34 [28], XPA is another key protein that can be lactylated by KAT5. By combining MS with site-directed mutagenesis screening, we identified six lysine residues (K141, K145, K217, K218, K221, and K222) in XPA as lactylation sites. These six lysine sites are located in the DNA-binding domain. The mutation of these six lysine residues to arginine (6KR) led to a significant reduction in XPA lactylation and abolished the ability of XPA to be lactylated by KAT5. Loss of XPA lactylation markedly reduced its DNA binding capacity following UV-induced damage, not respond to treatment with NALA, LDHi, or MG149. Although structural modeling and analysis reveal that XPA lactylation does not alter the conformation of its DNA-binding domain, the precise mechanism by which XPA lactylation facilitates its DNA binding remains unclear. Therefore, future structural studies of lactylated XPA may help elucidate the molecular basis underlying the enhanced DNA-binding affinity of XPA upon lactylation. Under UV irradiation, XPA lactylation increased the CPD repair rate, but XPA-6KR resulted in a much slower CPD repair rate. The cell survival assays revealed that XPA lactylation caused cell resistance to UV irradiation. Compared with XPA-WT cells, XPA-6KR cells were extremely sensitive to UV irradiation and did not respond to treatment with NALA or LDHi. These findings suggest that the lactylation of XPA mediated by KAT5 enhances the ability of XPA to bind DNA under UV irradiation, facilitating downstream signal transduction, accelerating the NER process, and finally leading to cell survival in response to UV irradiation. Therefore, XPA lactylation deficiency may enhance anti-tumor efficacy in combination with chemotherapeutic agents while potentially overcoming tumor chemoresistance.

Our results, consistent with previous studies [7, 39, 50], demonstrate that lactylation promotes DNA damage repair. However, what differs is that the referenced studies focus on the DNA damage repair of double-strand breaks, while the lactylation of XPA in our study regulated the NER of single-strand damage. Loss of XPA significantly augments the synthetic lethality between MK2 and p53, thereby potentiating antitumor response in combination with cisplatin chemotherapy [51]. Phosphorylation enhances the stability of the XPA protein by preventing its degradation through ubiquitination [46], thereby augmenting its functional capacity. Our observations indicate that neither NALA nor LDHi affects the stability of the XPA protein, implying that their regulatory effects on XPA may proceed through independent pathways. Previous research has shown that the deacetylation of XPA enhances its interaction with RPA32 [17], whereas lactylation does not impact the formation of the XPA–RPA32 complex, suggesting that these two post-translational modifications represent distinct regulatory mechanisms. PARylation facilitates the binding of XPA to DNA [18], and our findings consistently demonstrate that lactylation of XPA also enhances its association with DNA. PARylation predominantly targets serine residues on proteins, while lactylation modifies lysine residues. Considering that both modifications enhance XPA activity following UV-induced DNA damage, it is plausible that they may operate synergistically to amplify the DNA repair capacity mediated by XPA upon UV irradiation.​

In summary, this study links tumor metabolic reprogramming with DNA damage repair, elucidating the function and mechanism of lactylation in regulating UV damage repair. Our findings demonstrate that the lactylation of XPA enhances NER and leads to UV hyposensitivity, indicating that XPA can serve as a potential therapeutic target to reduce UV-induced damage. Therefore, developing new interventions targeting XPA lactylation may improve the therapeutic effects of radiotherapy and chemotherapy.

## Supplementary Material

gkag947_Supplemental_File

## Data Availability

The data underlying this article are available in the article and in its online supplementary material.
