# Supplementary material for: XPA lactylation is required for UV-induced DNA damage repair
Source: Nucleic Acids Res. 2026 Oct 7;54(18):gkag947. doi: 10.1093/nar/gkag947 (PMC13643169; doi:10.1093/nar/gkag947)
Supplement: Supplementary file 1 [file gkag947_supplemental_file.pdf]

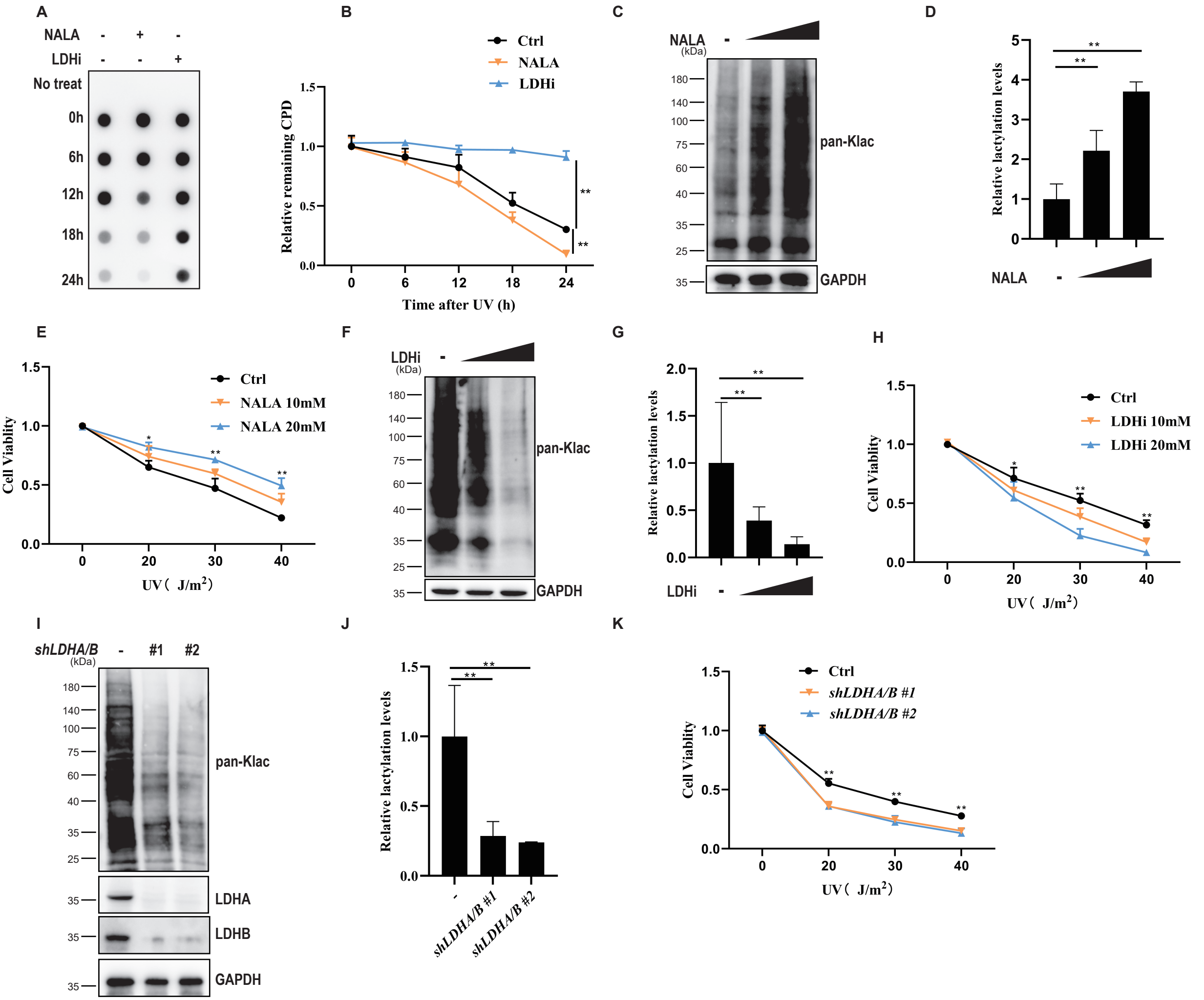

Figure S1 Induced lactylation promotes NER and UV resistance in cancer cells related to Figure 1

(A) HeLa cells were seeded into 6-well plates, treating with either LDHi or NALA for 24 h and harvested at different time points after 15 J/m² UV irradiation. Dlot-blot analysis was carried out, and UV-induced CPDs were detected with anti-CPD antibody.

(B) Quantification of the relative remaining CPD levels from three independent experiments related to (A).

(C) A549 cells were exposed to a gradient concentration of NALA or vehicle for 24 h, followed by lysis. Subsequently, immunoblotting was carried out using the indicated antibodies.

(D) Quantification of the lactylation levels from three independent experiments related to (A).

(E) A549 cells were treated with increasing doses of NALA UV irradiation, followed by 48 h recovery. CCK8 assay was performed as detailed in the Methods.

(F) A549 cells were exposed to a gradient concentration of LDHi or vehicle for 24 h, followed by lysis. Subsequently, immunoblotting was carried out using the indicated antibodies.

(G) Quantification of the lactylation levels from three independent experiments related to (D).

(H) A549 cells were treated with increasing doses of LDHi and UV irradiation, followed by 48 h recovery. CCK8 assay was performed as detailed in the Methods.

(I) A549 cells stably depleted of LDHA/B or control cells were lysed and then the immunoblotting was performed with indicated antibodies.

(J) Quantification of the lactylation levels from three independent experiments related to (I).

(K) Cells from (I) were utilized to perform CCK8 as indicated.

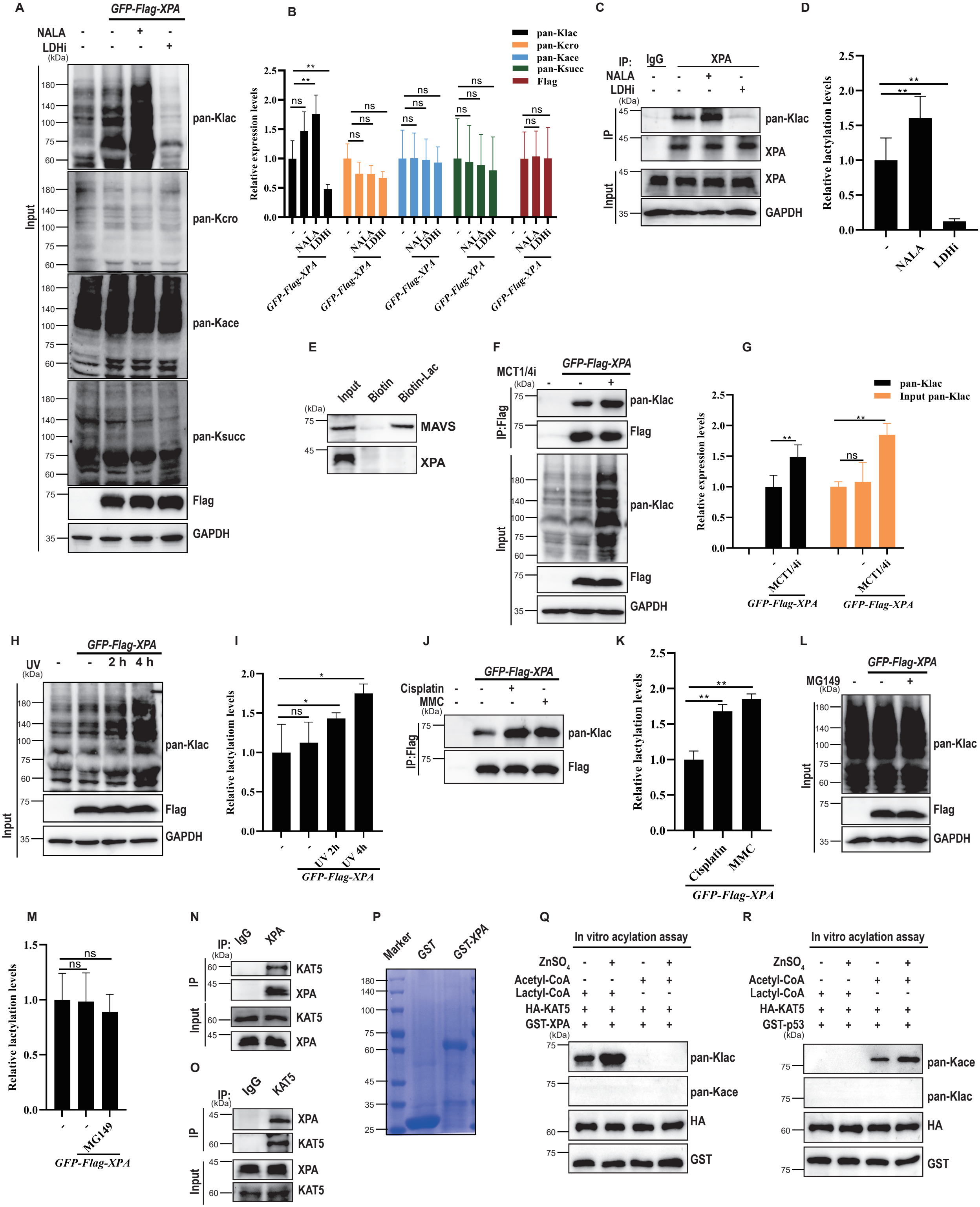
A
GFP-Flag-XPA
NALA
LDHi
(kDa)
Input
pan-Klac
pan-Kcro
pan-Kace
pan-Ksucc
Flag
GAPDH
B
pan-Klac
pan-Kcro
pan-Kace
pan-Ksucc
Flag
Relative expression levels
NALA
LDHi
GFP-Flag-XPA
ns
C
IP:
IgG
XPA
NALA
LDHi
(kDa)
IP
Input
pan-Klac
XPA
GAPDH
D
Relative lactylation levels
NALA
LDHi
E
Input
Biotin
Biotin-Lac
(kDa)
MAVS
XPA
F
MCT1/4i
GFP-Flag-XPA
(kDa)
IP:Flag
Input
pan-Klac
Flag
GAPDH
G
pan-Klac
Input pan-Klac
Relative expression levels
MCT1/4i
GFP-Flag-XPA
H
GFP-Flag-XPA
UV
2 h
4 h
(kDa)
Input
pan-Klac
Flag
GAPDH
I
Relative lactylatiom levels
UV 2h
UV 4h
GFP-Flag-XPA
J
GFP-Flag-XPA
Cisplatin
MMC
(kDa)
IP:Flag
pan-Klac
Flag
K
Relative lactylation levels
Cisplatin
MMC
GFP-Flag-XPA
L
GFP-Flag-XPA
MG149
(kDa)
Input
pan-Klac
Flag
GAPDH
M
Relative lactylation levels
MG149
GFP-Flag-XPA
N
IP:
IgG
XPA
(kDa)
IP
Input
KAT5
XPA
O
IP:
IgG
KAT5
(kDa)
IP
Input
XPA
KAT5
P
Marker
GST
GST-XPA
Q
In vitro acylation assay
ZnSO4
Acetyl-CoA
Lactyl-CoA
HA-KAT5
GST-XPA
(kDa)
pan-Klac
pan-Kace
HA
GST
R
In vitro acylation assay
ZnSO4
Acetyl-CoA
Lactyl-CoA
HA-KAT5
GST-p53
(kDa)
pan-Kace
pan-Klac
HA
GST

Figure S2 XPA is lactylated by KAT5 related to Figure 2

(A) Input were blotted with indicated antibodies related to Figure 2B.

(B) Quantification of the indicated protein levels from three independent experiments related to (A).

(C) HEK293T cells treated with either NALA or LDHi for 24 h before lysis. Cell lysates were immunoprecipitated with Protein A and G agarose beads pre-coupled with rabbit IgG (control), or anti-XPA antibody. The immunoprecipitates were then blotted with the indicated antibodies.

(D) Quantification of the lactylation levels from three independent experiments related to (C).

(E) HEK293T cells lysates were incubated with biotin-labeled lactate or biotin control, then blotted with the indicated antibodies.

(F) HEK293T cells were transfected with GFP-Flag-tagged XPA and treated with MCT1/4 inhibitor (20 μM) for 24 h before lysis. Cell lysates were immunoprecipitated with anti-Flag agarose beads, and western blot analysis was performed with indicated antibodies.

(G) Quantification of the indicated protein levels from three independent experiments related to (F).

(H) Input were blotted with indicated antibodies related to Figure 2G.

(I) Quantification of the lactylation levels from three independent experiments related to (H).

(J) HEK293T cells were transfected with GFP-Flag-tagged XPA and treated with cisplatin (20 μM) or MMC (10 μM) for 12 h before lysis. Cell lysates were immunoprecipitated with anti-Flag agarose beads, and western blot analysis was performed with indicated antibodies.

(K) Quantification of the lactylation levels from three independent experiments related to (J).

(L) Input were blotted with indicated antibodies related to Figure 2K.

(M) Quantification of the lactylation levels from three independent experiments related to (L).

(N) A549 cells were lysed and then purified with Protein A and G agarose beads pre-coupled with rabbit IgG (control) or anti-XPA antibody. Whole-cell lysis and the immunoprecipitates were then blotted with the indicated antibodies.

(O) A549 cells were lysed and then purified with Protein A and G agarose beads pre-coupled with rabbit IgG (control) or anti-KAT5 antibody. Whole-cell lysis and the immunoprecipitates were then blotted with the indicated antibodies.

(P) Purified GST and GST-XPA proteins were stained by Coomassie blue related to Figure 2Q.

(Q) GST-XPA proteins purified from E. Coli were incubated with HA-tagged KAT5 purified from HEK293T cells together with lactyl-CoA or acetyl-CoA in in vitro acylation buffer at 30 °C for 30 min. The reaction mixture was blotted with indicated antibodies.

(R) GST-p53 proteins purified from E. Coli were incubated with HA-tagged KAT5 purified from HEK293T cells together with lactyl-CoA or acetyl-CoA in in vitro acylation buffer at 30 °C for 30 min. The reaction mixture was blotted with indicated antibodies.

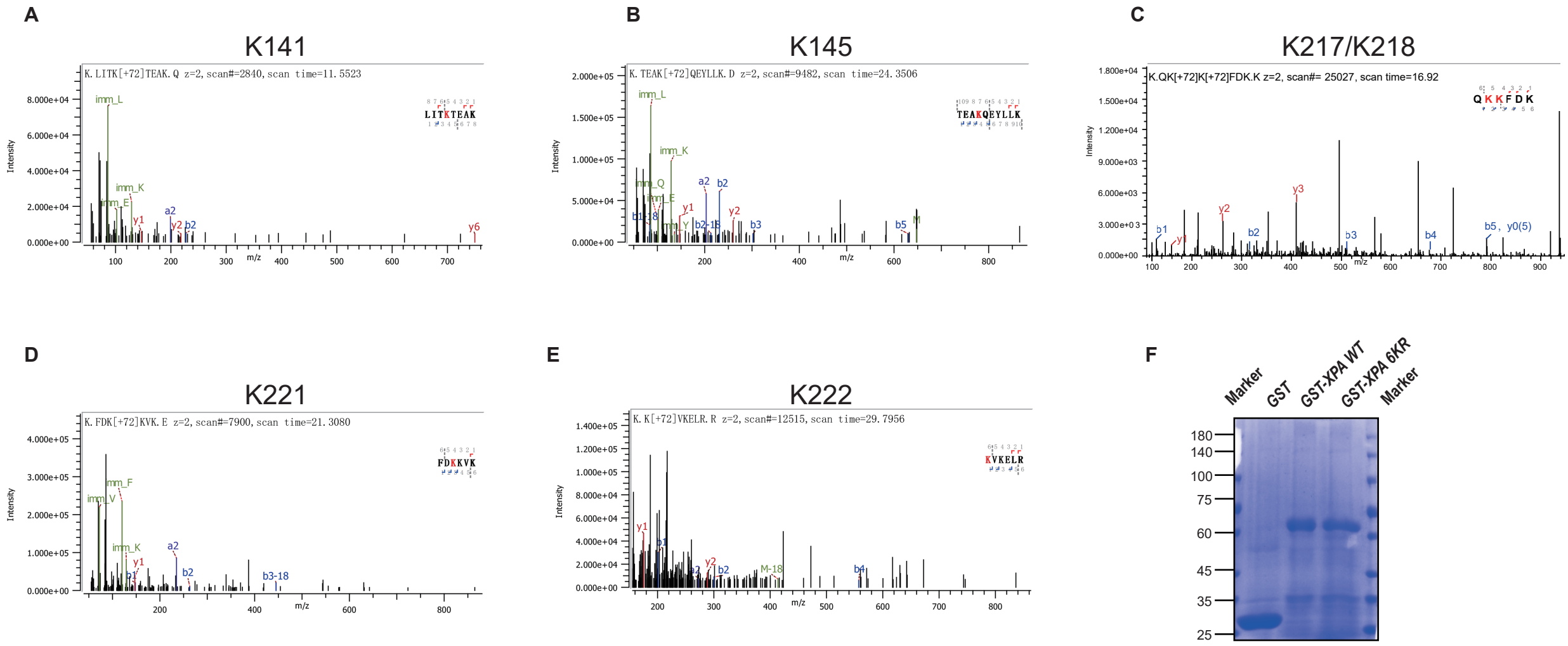

Figure S3 XPA is lactylated at six sites related to Figure 3

(A) Illustration of XPA lactylation at K141 derived from HEK293T cells identified by MS.

(B) Illustration of XPA lactylation at K145 derived from HEK293T cells identified by MS.

(C) Illustration of XPA lactylation at K217 and K218 derived from HEK293T cells identified by MS.

(D) Illustration of XPA lactylation at K221 derived from HEK293T cells identified by MS.

(E) Illustration of XPA lactylation at K222 derived from HEK293T cells identified by MS.

(F) Purified GST, GST-XPA, and GST-6KR proteins were stained by Coomassie blue related to Figure 3L.

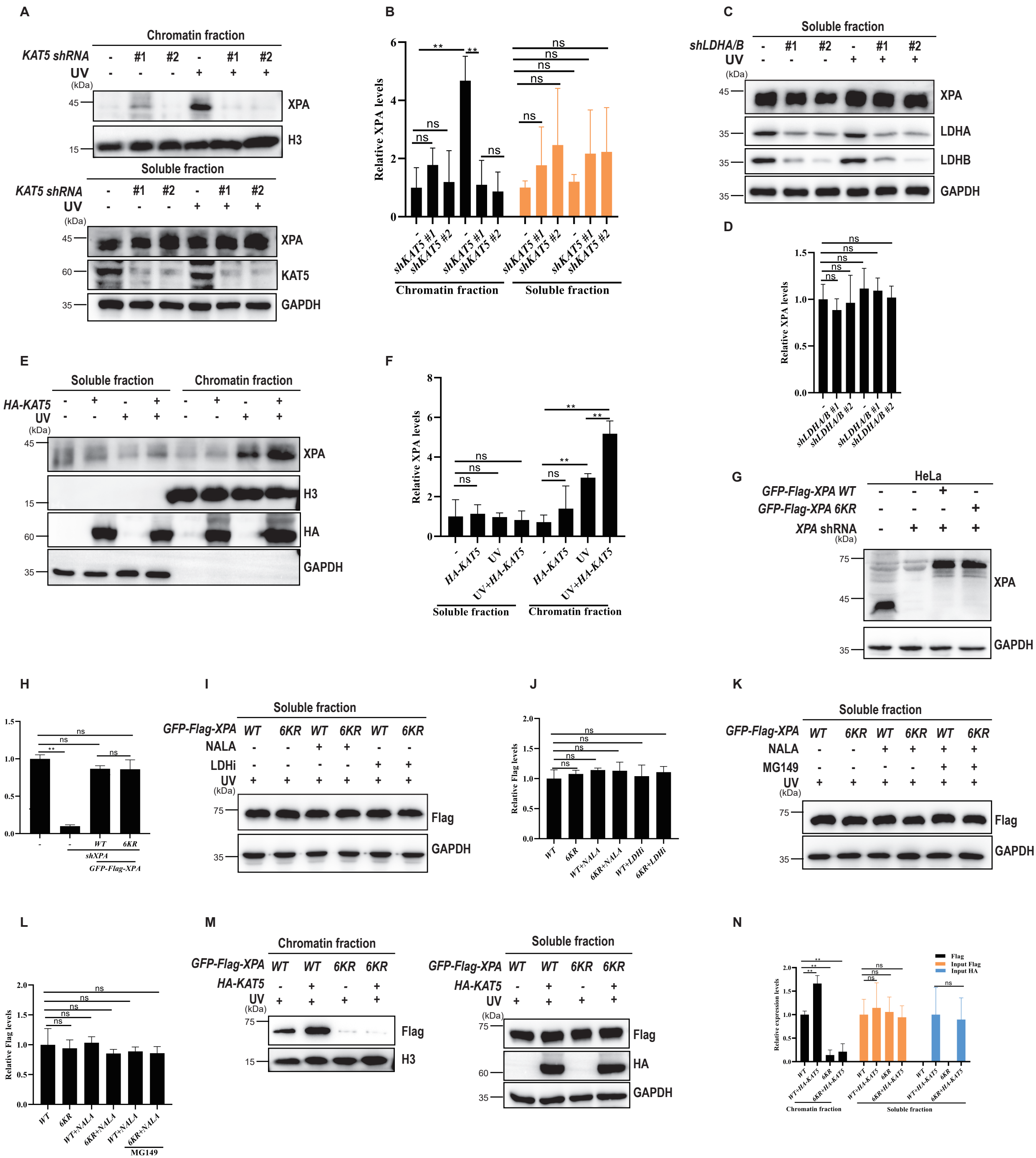
A
Chromatin fraction
KAT5 shRNA - #1 #2 - #1 #2
UV - - - + + +
(kDa)
45
15
XPA
H3
Soluble fraction
KAT5 shRNA - #1 #2 - #1 #2
UV - - - + + +
(kDa)
45
60
35
XPA
KAT5
GAPDH
B
Relative XPA levels
**
**
ns
ns
ns
- shKAT5 #1 shKAT5 #2 - shKAT5 #1 shKAT5 #2
Chromatin fraction
Soluble fraction
C
Soluble fraction
shLDHA/B - #1 #2 - #1 #2
UV - - - + + +
(kDa)
XPA
LDHA
LDHB
GAPDH
D
Relative XPA levels
ns
- shLDHA/B #1 shLDHA/B #2 - shLDHA/B #1 shLDHA/B #2
E
Soluble fraction
Chromatin fraction
HA-KAT5 - + - + - + - +
UV - - + + - - + +
(kDa)
XPA
H3
HA
GAPDH
F
Relative XPA levels
ns
**
- HA-KAT5 UV UV+HA-KAT5 - HA-KAT5 UV UV+HA-KAT5
Soluble fraction
Chromatin fraction
G
HeLa
GFP-Flag-XPA WT - - + -
GFP-Flag-XPA 6KR - - - +
XPA shRNA - + + +
(kDa)
XPA
GAPDH
H
Relative XPA levels
**
ns
WT 6KR
shXPA
GFP-Flag-XPA
I
Soluble fraction
GFP-Flag-XPA WT 6KR WT 6KR WT 6KR
NALA - - + + - -
LDHi - - - - + +
UV + + + + + +
(kDa)
Flag
GAPDH
J
Relative Flag levels
ns
WT 6KR WT+NALA 6KR+NALA WT+LDHi 6KR+LDHi
K
Soluble fraction
GFP-Flag-XPA WT 6KR WT 6KR WT 6KR
NALA - - + + + +
MG149 - - - - + +
UV + + + + + +
(kDa)
Flag
GAPDH
L
Relative Flag levels
ns
WT 6KR WT+NALA 6KR+NALA WT+NALA 6KR+NALA
MG149
M
Chromatin fraction
GFP-Flag-XPA WT WT 6KR 6KR
HA-KAT5 - + - +
UV + + + +
(kDa)
Flag
H3
Soluble fraction
GFP-Flag-XPA WT WT 6KR 6KR
HA-KAT5 - + - +
UV + + + +
(kDa)
Flag
HA
GAPDH
N
Relative expression levels
Flag
Input Flag
Input HA
**
ns
WT WT+HA-KAT5 6KR 6KR+HA-KAT5
Chromatin fraction
Soluble fraction

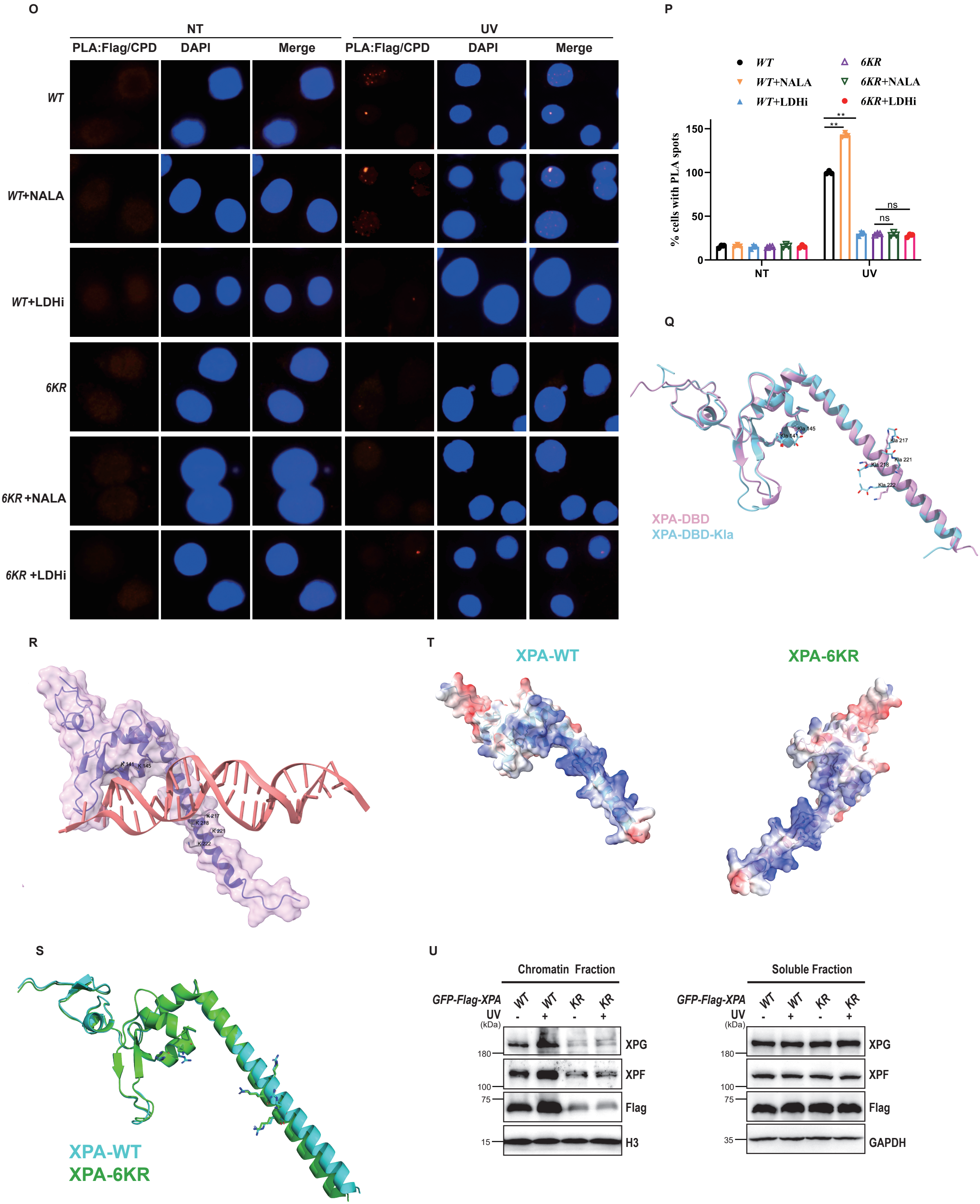
O
NT
UV
PLA:Flag/CPD
DAPI
Merge
PLA:Flag/CPD
DAPI
Merge
WT
WT+NALA
WT+LDHi
6KR
6KR +NALA
6KR +LDHi
P
WT
WT+NALA
WT+LDHi
6KR
6KR+NALA
6KR+LDHi
% cells with PLA spots
150
100
50
0
**
**
ns
ns
NT
UV
Q
Kla 141
Kla 145
Kla 217
Kla 221
Kla 218
Kla 222
XPA-DBD
XPA-DBD-Kla
R
K 141
K 145
K 217
K 218
K 221
K 222
T
XPA-WT
XPA-6KR
S
XPA-WT
XPA-6KR
U
Chromatin Fraction
Soluble Fraction
GFP-Flag-XPA
WT WT KR KR
UV
- + - +
(kDa)
180
100
75
15
35
XPG
XPF
Flag
H3
GAPDH

Figure S4 Lactylation enhances the ability of XPA to bind DNA related to Figure 4

(A) Depleted of KAT5 in HeLa cells or control cells were irradiated with UV (60 J/m²) and then allowed to recover for 4 h. Cell lysates were divided into soluble fraction and chromatin fraction. These two components were blotted with indicated antibodies.

(B) Quantification of the XPA levels from three independent experiments related to (A).

(C) Soluble fraction was blotted with indicated antibodies related to Figure 4K.

(D) Quantification of the XPA levels from three independent experiments related to (C).

(E) HeLa cells were transfected with HA-tagged KAT5, and then cells were irradiated with UV (60 J/m²) and allowed to recover for 4 h. The cell lysates were divided into soluble fraction and chromatin fraction. These two components were blotted with indicated antibodies.

(F) Quantification of the XPA levels from three independent experiments related to (E).

(G) Western blot of HeLa cells as indicated.

(H) Quantification of the XPA levels from three independent experiments related to (G).

(I) Soluble fraction was blotted with indicated antibodies related to Figure 4O.

(J) Quantification of the GFP-Flag-XPA levels from three independent experiments related to (I).

(K) Soluble fraction was blotted with indicated antibodies related to Figure 4Q.

(L) Quantification of the GFP-Flag-XPA levels from three independent experiments related to (K).

(M) HeLa cells were transfected with GFP-Flag-tagged XPA WT or XPA 6KR together with HA-tagged KAT5. The cells were irradiated with UV (60 J/m²) and allowed to recover for 4 h. The cell lysates were divided into soluble fraction and chromatin fraction. These two components were blotted with indicated antibodies.

(N) Quantification of the indicated protein levels from three independent experiments related to (M).

(O) HeLa cells stably expressing XPA WT or XPA 6KR were treated with NALA or LDHi for 24 h. Then, cells were irradiated with UV through 5-micron filters and allowed to recover for 3 h before subjecting to PLA with anti-Flag and anti-CPD antibodies.

(P) The displayed results represent the averages of three separate experiments and were presented as mean values ± SEM normalized to the WT group treated with UV irradiation. More than 200 cells were counted for each condition. NT: not treated.

(Q) Structural superimposition of the DNA-binding domain (DBD) of wild-type XPA (XPA-DBD) and lactylated XPA (XPA-DBD-Kla). Kla: lactylation.

(R) Structural analysis of the XPA-DNA complex.

(S) Structural superimposition analysis of the XPA-WT and XPA-6KR mutant.

(T) Electrostatics analysis of the XPA-WT and XPA-6KR mutant based on the structural. blue: positive charge, red: negative charge.

(U) HeLa cells stably expressing XPA WT or XPA 6KR were irradiated with UV (60 J/m²) and allowed to recover for 4 h. The cell lysates were divided into soluble fraction and chromatin fraction. These two components were blotted with indicated antibodies.

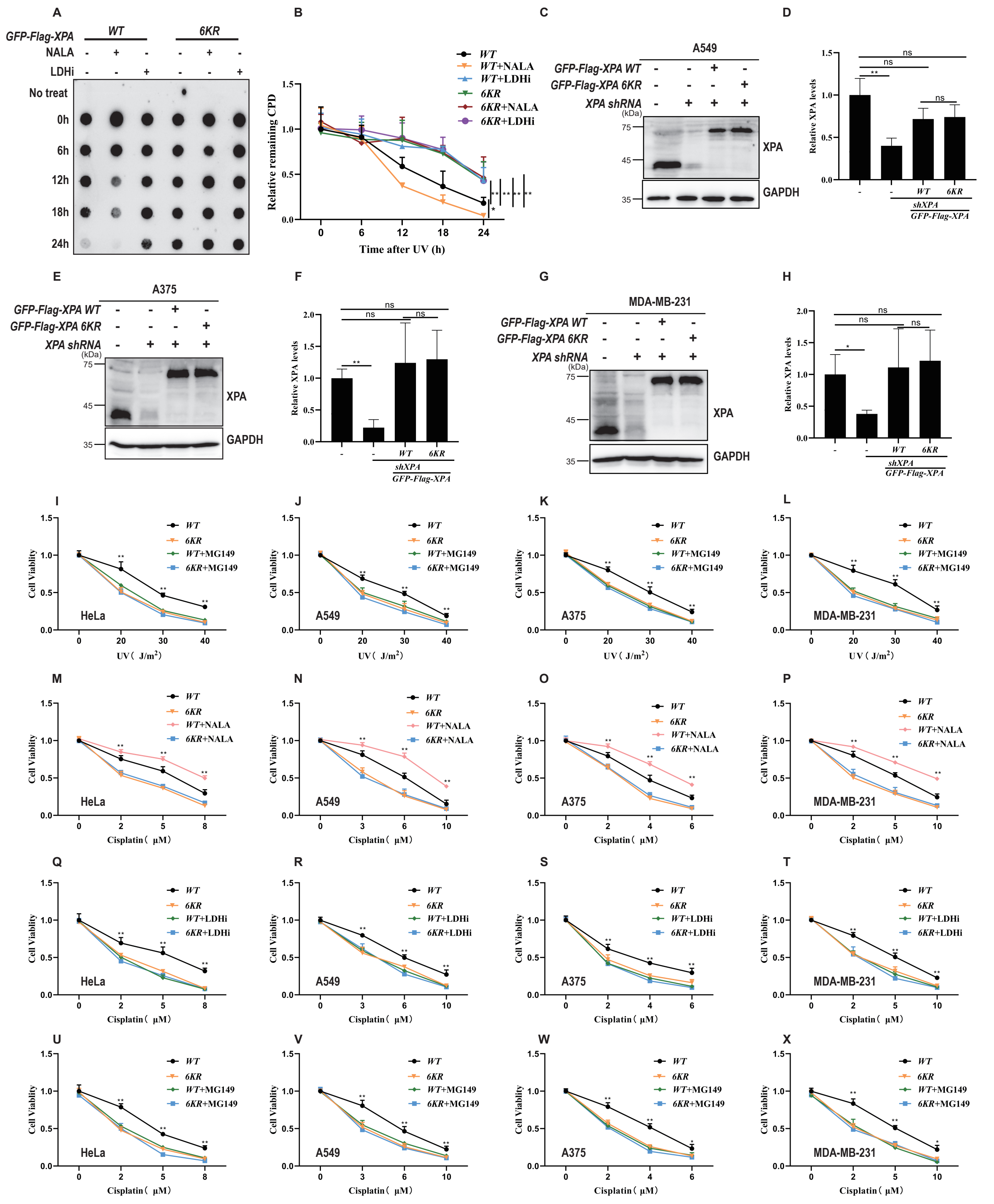
A
GFP-Flag-XPA
WT
6KR
NALA
LDHi
No treat
0h
6h
12h
18h
24h
B
Relative remaining CPD
Time after UV (h)
WT
WT+NALA
WT+LDHi
6KR
6KR+NALA
6KR+LDHi
C
A549
GFP-Flag-XPA WT
GFP-Flag-XPA 6KR
XPA shRNA
(kDa)
75
45
35
XPA
GAPDH
D
Relative XPA levels
ns
shXPA
GFP-Flag-XPA
E
A375
F
G
MDA-MB-231
H
I
J
K
L
Cell Viability
HeLa
A549
A375
MDA-MB-231
UV（ J/m²）
WT+MG149
6KR+MG149
M
N
O
P
Cisplatin（ μM）
Q
R
S
T
U
V
W
X

Figure S5 XPA lactylation promotes NER and cell survival under UV-induced damage related to Figure 5

(A) HeLa cells stbaly expressing XPA-WT or XPA-6KR were seeded into 6-well plates, treating with either LDHi or NALA for 24 h and harvested at different time points after 15 J/m² UV irradiation. Dlot-blot analysis was carried out, and UV-induced CPDs were detected with anti-CPD antibody.

(B) Quantification of the relative remaining CPD levels from three independent experiments related to (A).

(C) Western blot with indicated antibodies in A549 cells stably expressing GFP-Flag-tagged XPA WT or GFP-Flag-tagged XPA 6KR.

(D) Quantification of the XPA levels from three independent experiments related to (A).

(E) Western blot with indicated antibodies in A375 cells stably expressing GFP-Flag-tagged XPA WT or GFP-Flag-tagged XPA 6KR.

(F) Quantification of the XPA from three independent experiments related to (C).

(G) Western blot with indicated antibodies in MDA-MB-231 cells stably expressing GFP-Flag-tagged XPA WT or GFP-Flag-tagged XPA 6KR.

(H) Quantification of the XPA from three independent experiments related to (E).

(I-L) Cell viability was assessed in XPA-WT or 6KR HeLa (I), A549 (J), A375 (K) and MDA-MB-231 (L) cells treated with MG149 under UV irradiation.

(M-P) Cell viability was assessed in XPA-WT or 6KR HeLa (M), A549 (N), A375 (O) and MDA-MB-231 (P) cells treated with NALA under cisplatin.

(Q-T) Cell viability was assessed in XPA-WT or 6KR HeLa (Q), A549 (R), A375 (S) and MDA-MB-231 (T) cells treated with LDHi under cisplatin.

(U-X) Cell viability was assessed in XPA-WT or 6KR HeLa (U), A549 (V), A375 (W) and MDA-MB-231 (X) cells treated with MG149 under cisplatin.

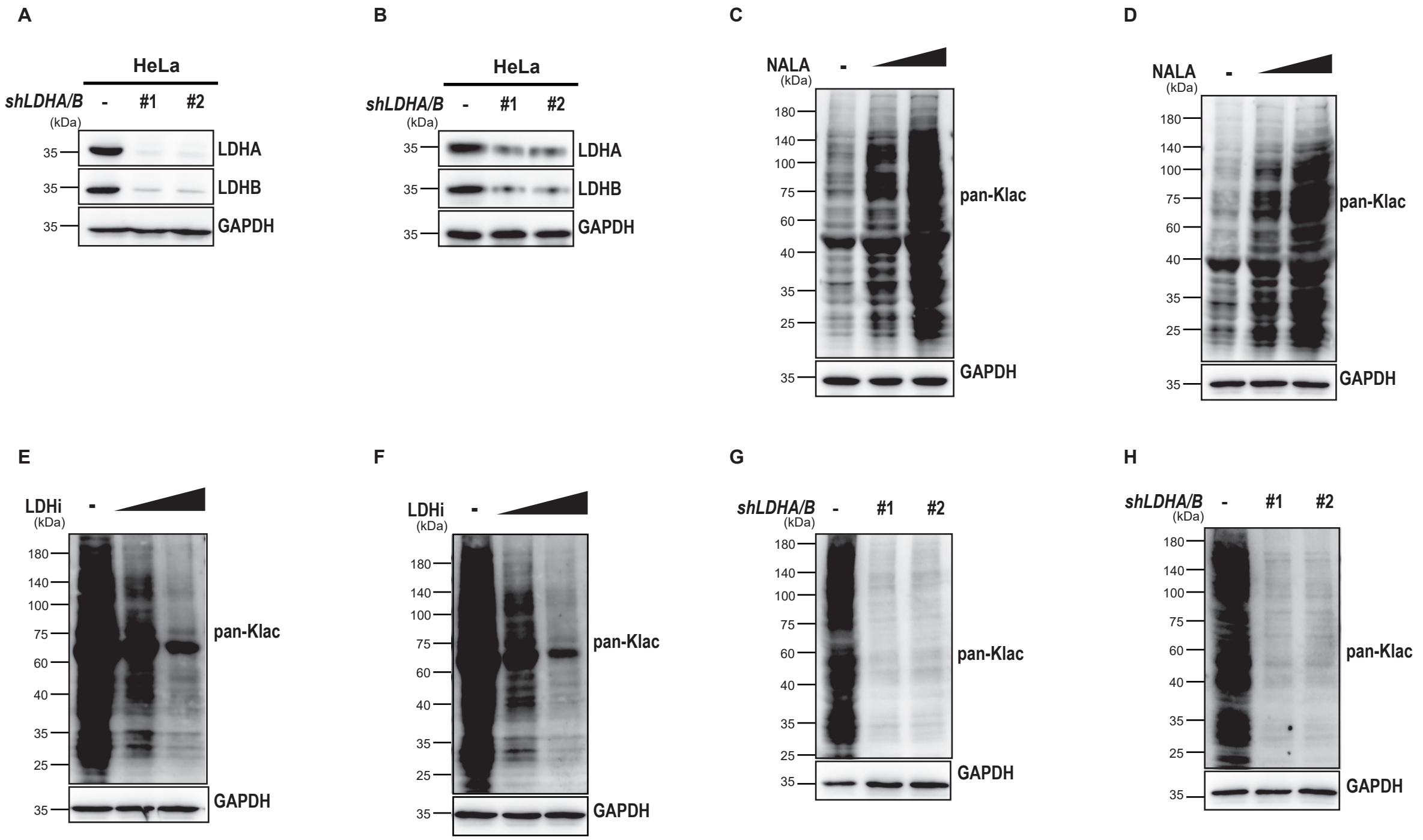

Figure S6 Induced lactylation promotes NER and UV resistance in cancer cells related to Figure 1

(A-B) Two additional independent replicate experiments for Figure 1C.

(C-D) Two additional independent replicate experiments for Figure 1G.

(E-F) Two additional independent replicate experiments for Figure 1J.

(G-H) Two additional independent replicate experiments for Figure 1M.

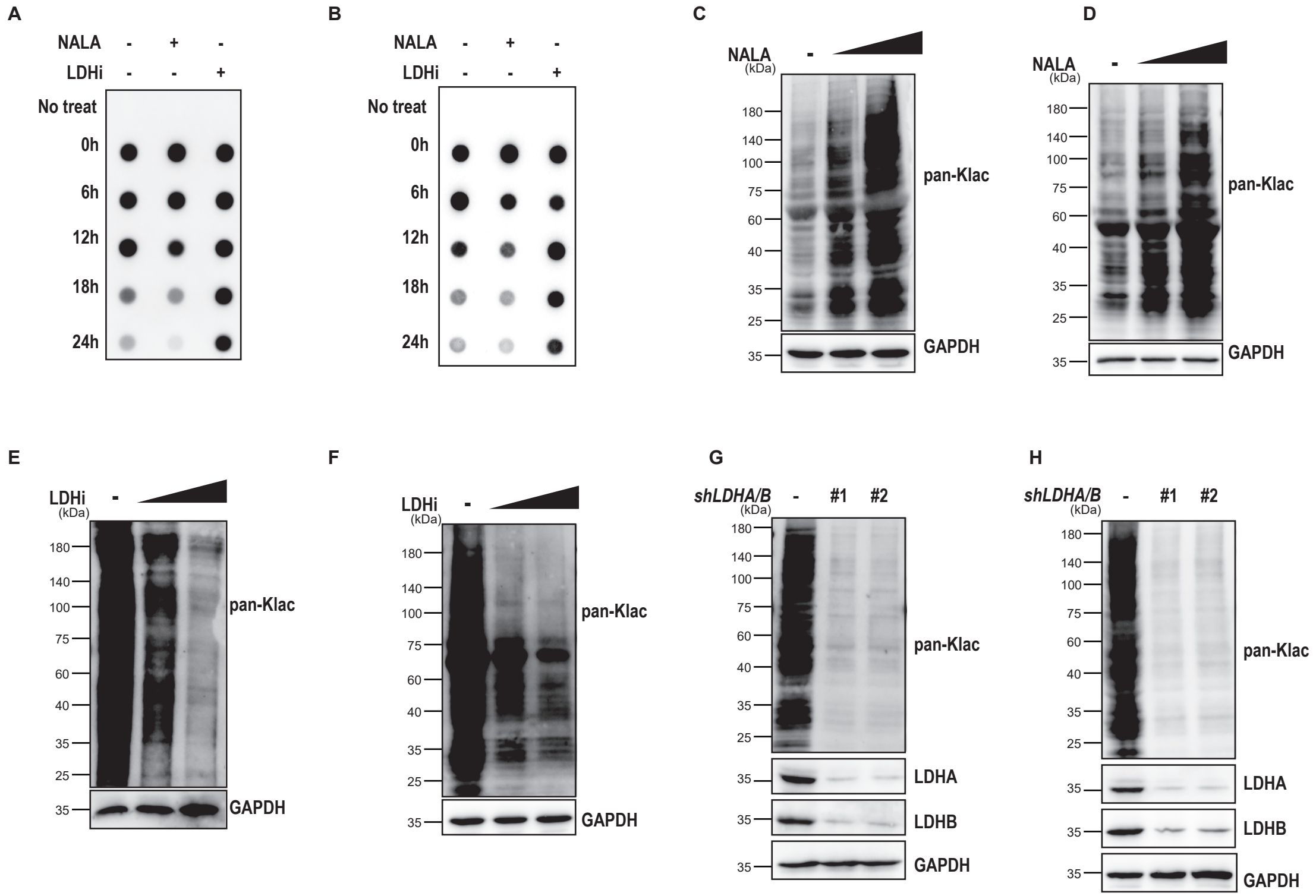

Figure S7 Induced lactylation promotes NER and UV resistance in cancer cells related to Figure S1

(A-B) Two additional independent replicate experiments for Figure S1A.

(C-D) Two additional independent replicate experiments for Figure S1C.

(E-F) Two additional independent replicate experiments for Figure S1F.

(G-H) Two additional independent replicate experiments for Figure S1I.

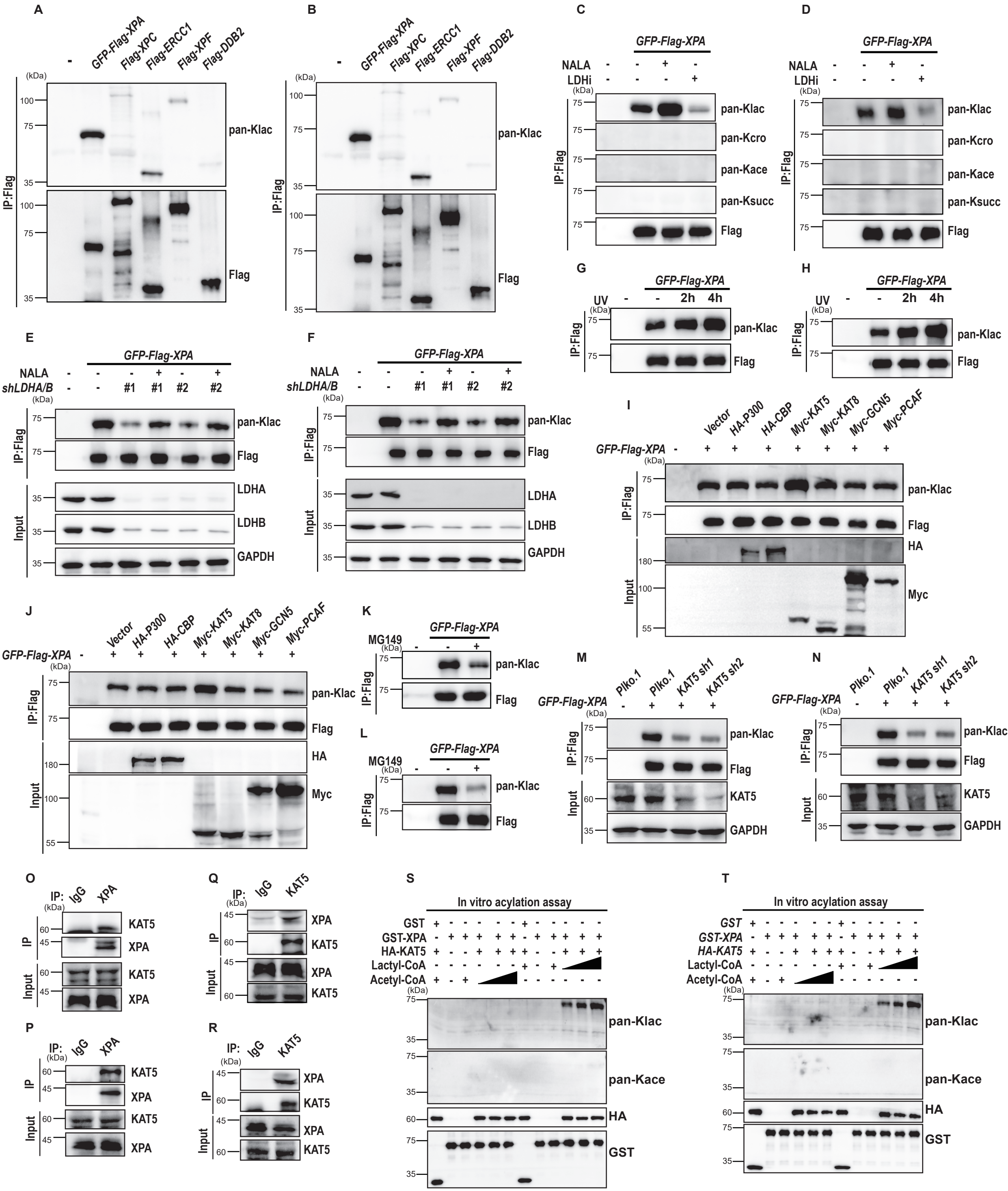

A
B
GFP-Flag-XPA
Flag-XPC
Flag-ERCC1
Flag-XPF
Flag-DDB2
(kDa)
IP:Flag
pan-Klac
Flag
C
D
NALA
LDHi
pan-Kcro
pan-Kace
pan-Ksucc
E
F
shLDHA/B
#1
#2
Input
LDHA
LDHB
GAPDH
G
H
UV
2h
4h
I
J
Vector
HA-P300
HA-CBP
Myc-KAT5
Myc-KAT8
Myc-GCN5
Myc-PCAF
HA
Myc
K
L
MG149
M
N
Plko.1
KAT5 sh1
KAT5 sh2
KAT5
O
P
Q
R
IP:
IgG
XPA
IP
S
T
In vitro acylation assay
GST
GST-XPA
HA-KAT5
Lactyl-CoA
Acetyl-CoA

Figure S8 XPA is lactylated by KAT5 related to Figure 2

(A-B) Two additional independent replicate experiments for Figure 2A.

(C-D) Two additional independent replicate experiments for Figure 2C.

(E-F) Two additional independent replicate experiments for Figure 2E.

(G-H) Two additional independent replicate experiments for Figure 2G.

(I-J) Two additional independent replicate experiments for Figure 2I.

(K-L) Two additional independent replicate experiments for Figure 2K.

(M-N) Two additional independent replicate experiments for Figure 2M.

(O-P) Two additional independent replicate experiments for Figure 2O.

(Q-R) Two additional independent replicate experiments for Figure 2P.

(S-T) Two additional independent replicate experiments for Figure 2Q.

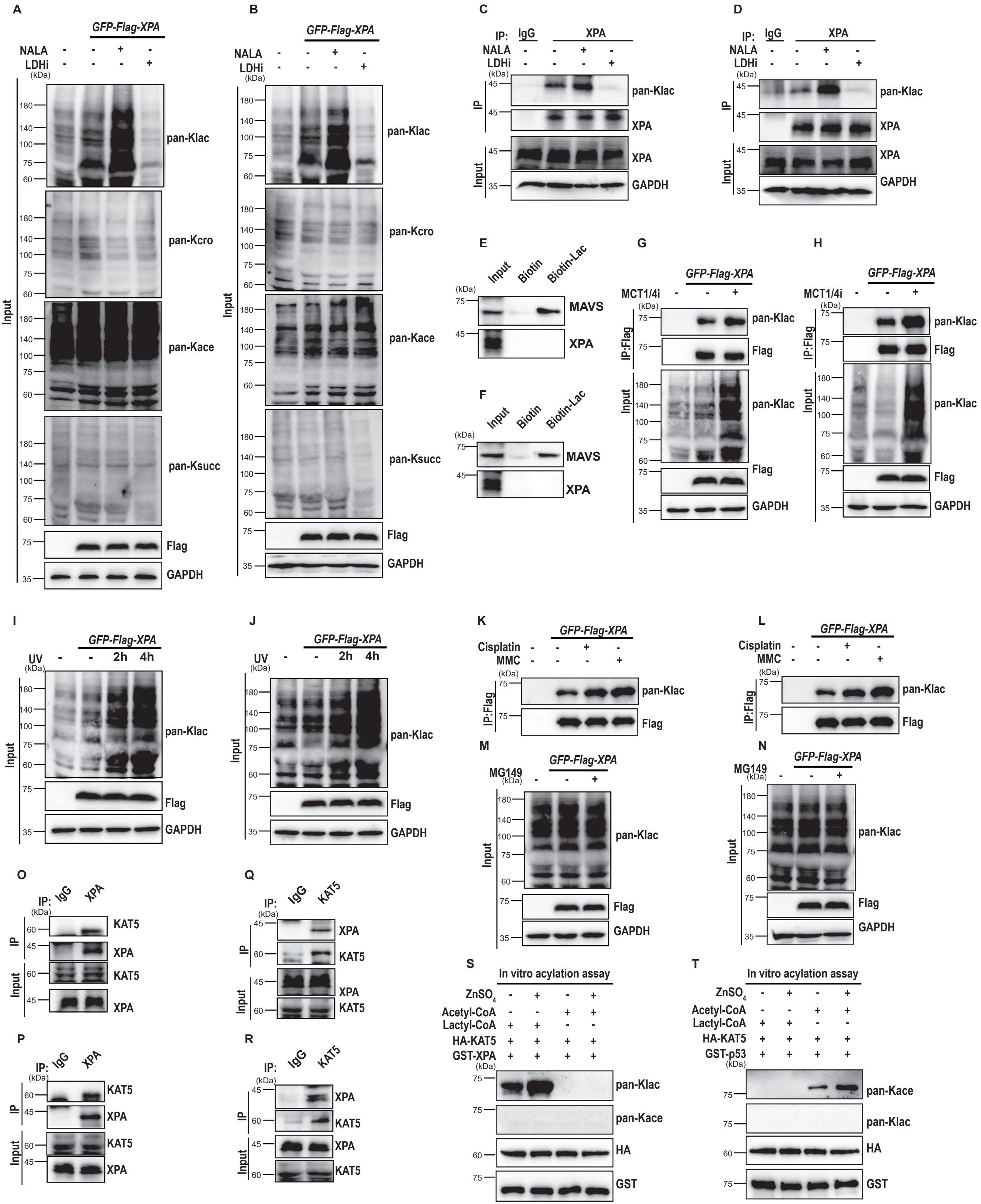
A
GFP-Flag-XPA
NALA
LDHi
(kDa)
pan-Klac
pan-Kcro
pan-Kace
pan-Ksucc
Flag
GAPDH
Input
B
GFP-Flag-XPA
NALA
LDHi
(kDa)
pan-Klac
pan-Kcro
pan-Kace
pan-Ksucc
Flag
GAPDH
Input
C
IP: IgG XPA
NALA
LDHi
(kDa)
IP
pan-Klac
XPA
Input
XPA
GAPDH
D
IP: IgG XPA
NALA
LDHi
(kDa)
IP
pan-Klac
XPA
Input
XPA
GAPDH
E
Input Biotin Biotin-Lac
(kDa)
MAVS
XPA
F
Input Biotin Biotin-Lac
(kDa)
MAVS
XPA
G
GFP-Flag-XPA
MCT1/4i
(kDa)
IP:Flag
pan-Klac
Flag
Input
pan-Klac
Flag
GAPDH
H
GFP-Flag-XPA
MCT1/4i
(kDa)
IP:Flag
pan-Klac
Flag
Input
pan-Klac
Flag
GAPDH
I
GFP-Flag-XPA
UV - - 2h 4h
(kDa)
Input
pan-Klac
Flag
GAPDH
J
GFP-Flag-XPA
UV - - 2h 4h
(kDa)
Input
pan-Klac
Flag
GAPDH
K
GFP-Flag-XPA
Cisplatin
MMC
(kDa)
IP:Flag
pan-Klac
Flag
L
GFP-Flag-XPA
Cisplatin
MMC
(kDa)
IP:Flag
pan-Klac
Flag
M
GFP-Flag-XPA
MG149
(kDa)
Input
pan-Klac
Flag
GAPDH
N
GFP-Flag-XPA
MG149
(kDa)
Input
pan-Klac
Flag
GAPDH
O
IP: IgG XPA
(kDa)
IP
KAT5
XPA
Input
KAT5
XPA
P
IP: IgG XPA
(kDa)
IP
KAT5
XPA
Input
KAT5
XPA
Q
IP: IgG KAT5
(kDa)
IP
XPA
KAT5
Input
XPA
KAT5
R
IP: IgG KAT5
(kDa)
IP
XPA
KAT5
Input
XPA
KAT5
S
In vitro acylation assay
ZnSO4
Acetyl-CoA
Lactyl-CoA
HA-KAT5
GST-XPA
(kDa)
pan-Klac
pan-Kace
HA
GST
T
In vitro acylation assay
ZnSO4
Acetyl-CoA
Lactyl-CoA
HA-KAT5
GST-p53
(kDa)
pan-Kace
pan-Klac
HA
GST

Figure S9 XPA is lactylated by KAT5 related to Figure S2

(A-B) Two additional independent replicate experiments for Figure S2A.

(C-D) Two additional independent replicate experiments for Figure S2C.

(E-F) Two additional independent replicate experiments for Figure S2E.

(G-H) Two additional independent replicate experiments for Figure S2F.

(I-J) Two additional independent replicate experiments for Figure S2H.

(K-L) Two additional independent replicate experiments for Figure S2J.

(M-N) Two additional independent replicate experiments for Figure S2L.

(O-P) Two additional independent replicate experiments for Figure S2N.

(Q-R) Two additional independent replicate experiments for Figure S2O.

(S) Additional independent replicate experiments for Figure S2Q.

(T) Additional independent replicate experiments for Figure S2R.

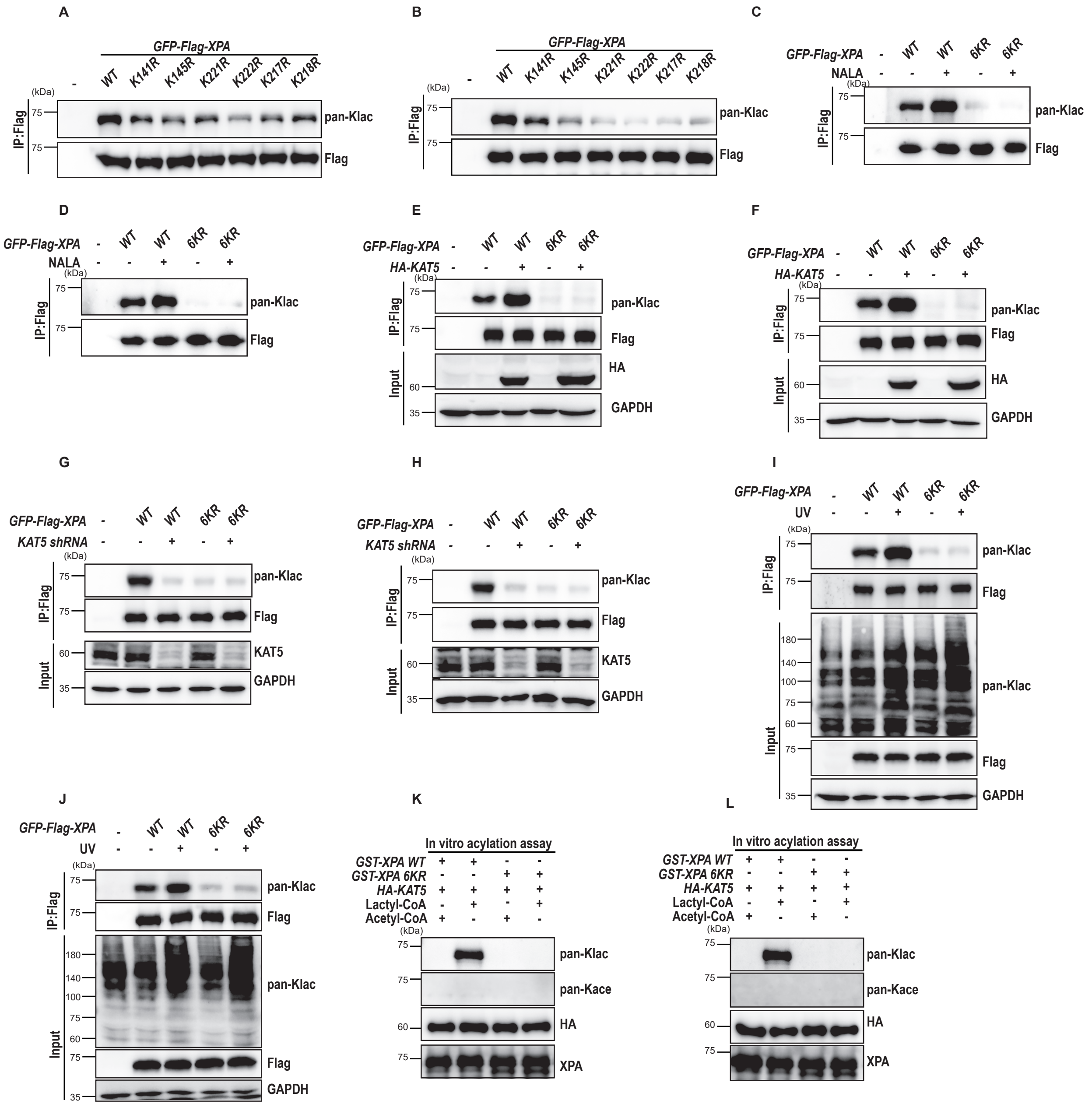

Figure S10 XPA is lactylated at six sites related to Figure 3

(A-B) Two additional independent replicate experiments for Figure 3B.

(C-D) Two additional independent replicate experiments for Figure 3D.

(E-F) Two additional independent replicate experiments for Figure 3F.

(G-H) Two additional independent replicate experiments for Figure 3H.

(I-J) Two additional independent replicate experiments for Figure 3J

(K-L) Two additional independent replicate experiments for Figure 3L.

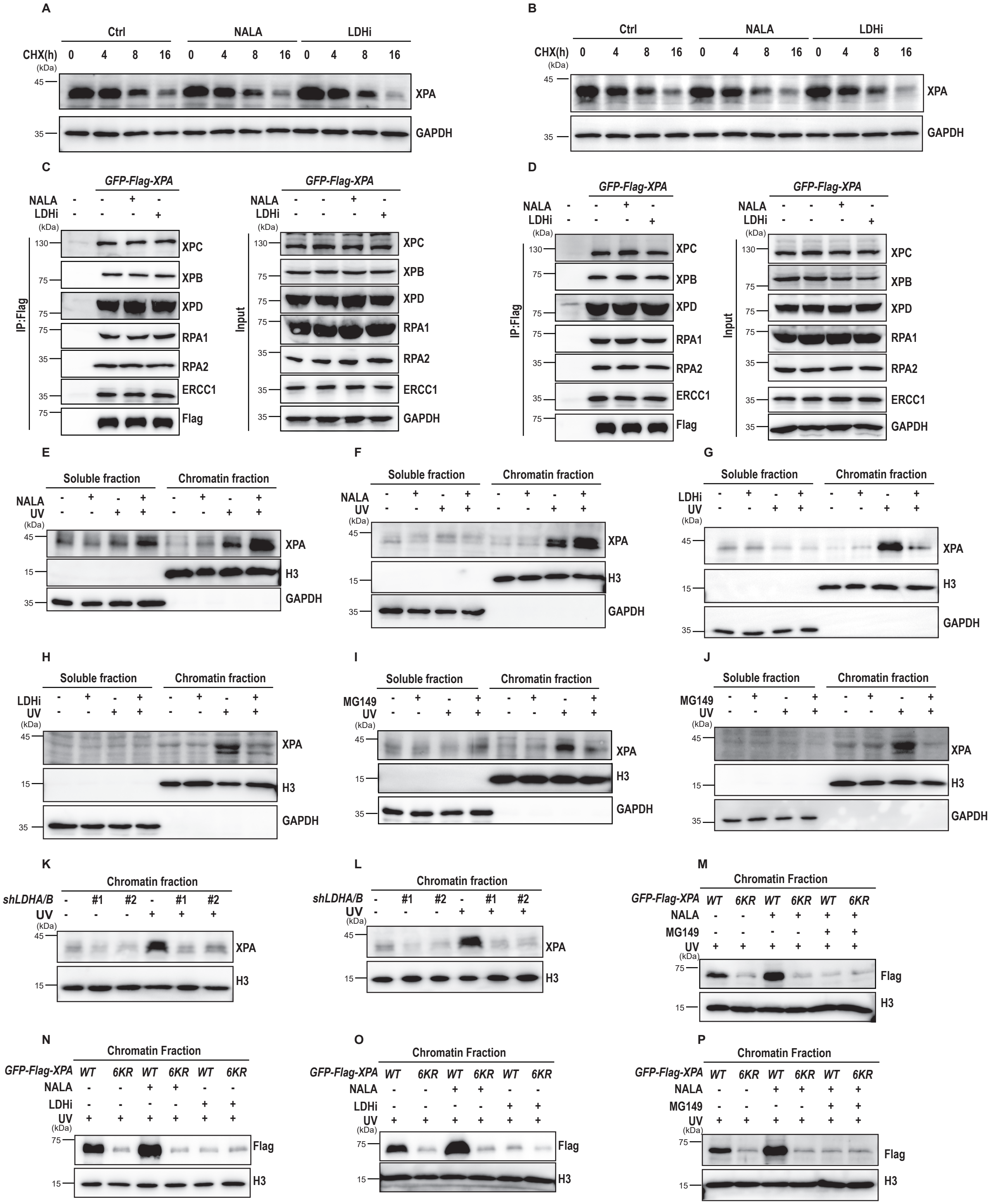
A
Ctrl
NALA
LDHi
CHX(h)
0
4
8
16
(kDa)
45
35
XPA
GAPDH
B
Ctrl
NALA
LDHi
CHX(h)
0
4
8
16
(kDa)
45
35
XPA
GAPDH
C
GFP-Flag-XPA
NALA
LDHi
(kDa)
IP:Flag
130
75
35
XPC
XPB
XPD
RPA1
RPA2
ERCC1
Flag
Input
XPC
XPB
XPD
RPA1
RPA2
ERCC1
GAPDH
D
GFP-Flag-XPA
NALA
LDHi
(kDa)
IP:Flag
XPC
XPB
XPD
RPA1
RPA2
ERCC1
Flag
Input
XPC
XPB
XPD
RPA1
RPA2
ERCC1
GAPDH
E
Soluble fraction
Chromatin fraction
NALA
UV
(kDa)
45
15
35
XPA
H3
GAPDH
F
Soluble fraction
Chromatin fraction
NALA
UV
XPA
H3
GAPDH
G
Soluble fraction
Chromatin fraction
LDHi
UV
XPA
H3
GAPDH
H
Soluble fraction
Chromatin fraction
LDHi
UV
XPA
H3
GAPDH
I
Soluble fraction
Chromatin fraction
MG149
UV
XPA
H3
GAPDH
J
Soluble fraction
Chromatin fraction
MG149
UV
XPA
H3
GAPDH
K
Chromatin fraction
shLDHA/B
#1
#2
UV
XPA
H3
L
Chromatin fraction
shLDHA/B
#1
#2
UV
XPA
H3
M
Chromatin Fraction
GFP-Flag-XPA
WT
6KR
NALA
MG149
UV
(kDa)
75
15
Flag
H3
N
Chromatin Fraction
GFP-Flag-XPA
WT
6KR
NALA
LDHi
UV
Flag
H3
O
Chromatin Fraction
GFP-Flag-XPA
WT
6KR
NALA
LDHi
UV
Flag
H3
P
Chromatin Fraction
GFP-Flag-XPA
WT
6KR
NALA
MG149
UV
Flag
H3

Figure S11 XPA is lactylated by KAT5 related to Figure 4

(A-B) Two additional independent replicate experiments for Figure 4A.
(C-D) Two additional independent replicate experiments for Figure 4C.
(E-F) Two additional independent replicate experiments for Figure 4E.
(G-H) Two additional independent replicate experiments for Figure 4G.
(I-J) Two additional independent replicate experiments for Figure 4I.
(K-L) Two additional independent replicate experiments for Figure 4K.
(M-N) Two additional independent replicate experiments for Figure 4O.
(O-P) Two additional independent replicate experiments for Figure 4Q.

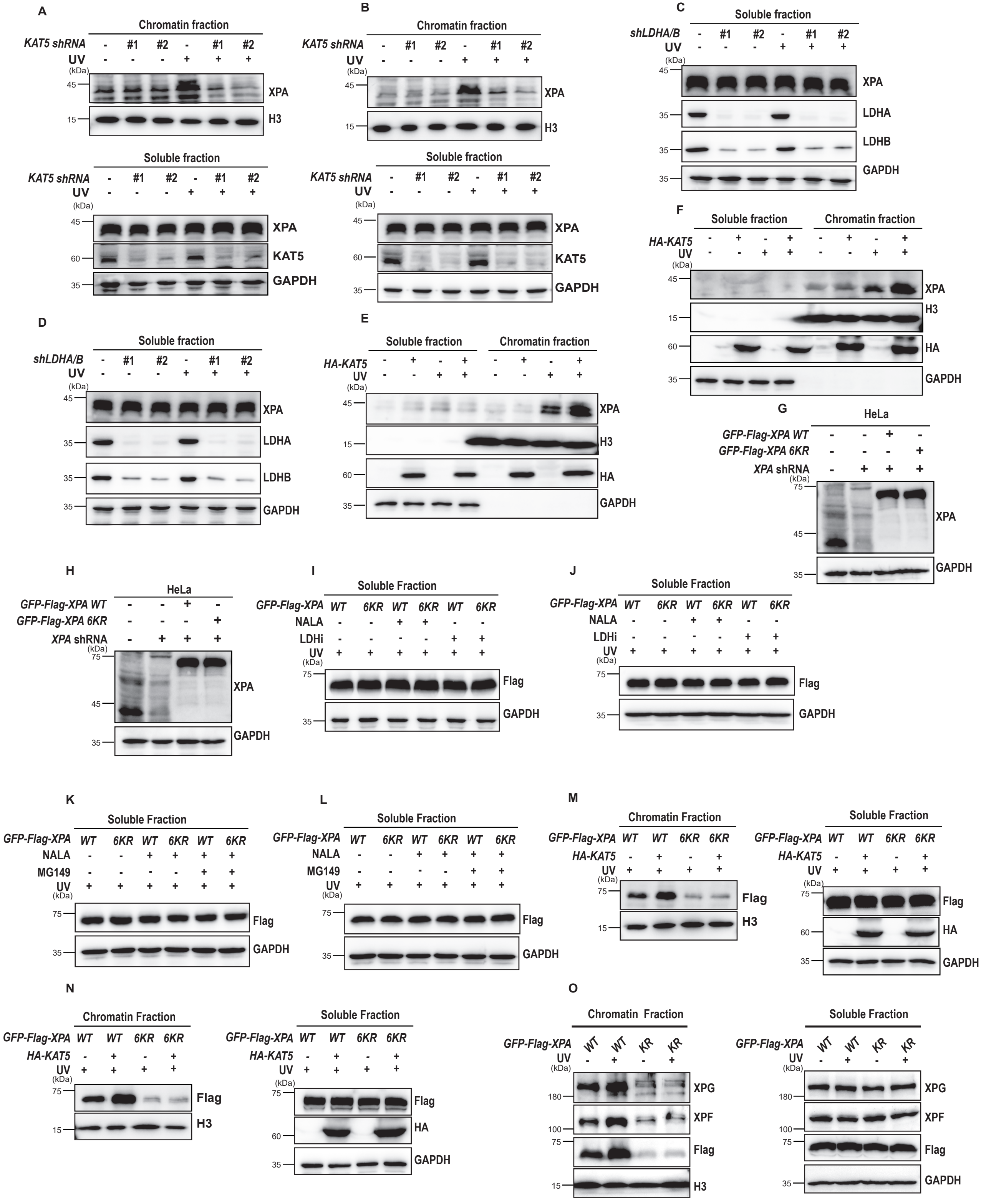
A
Chromatin fraction
KAT5 shRNA - #1 #2 - #1 #2
UV - - - + + +
(kDa)
45
XPA
15
H3
Soluble fraction
KAT5 shRNA - #1 #2 - #1 #2
UV - - - + + +
(kDa)
45
XPA
60
KAT5
35
GAPDH
B
Chromatin fraction
KAT5 shRNA - #1 #2 - #1 #2
UV - - - + + +
(kDa)
45
XPA
15
H3
Soluble fraction
KAT5 shRNA - #1 #2 - #1 #2
UV - - - + + +
(kDa)
45
XPA
60
KAT5
35
GAPDH
C
Soluble fraction
shLDHA/B - #1 #2 - #1 #2
UV - - - + + +
(kDa)
45
XPA
35
LDHA
35
LDHB
35
GAPDH
D
Soluble fraction
shLDHA/B - #1 #2 - #1 #2
UV - - - + + +
(kDa)
45
XPA
35
LDHA
35
LDHB
35
GAPDH
E
Soluble fraction
Chromatin fraction
HA-KAT5 - + - + - + - +
UV - - + + - - + +
(kDa)
45
XPA
15
H3
60
HA
35
GAPDH
F
Soluble fraction
Chromatin fraction
HA-KAT5 - + - + - + - +
UV - - + + - - + +
(kDa)
45
XPA
15
H3
60
HA
35
GAPDH
G
HeLa
GFP-Flag-XPA WT - - + -
GFP-Flag-XPA 6KR - - - +
XPA shRNA - + + +
(kDa)
75
45
XPA
35
GAPDH
H
HeLa
GFP-Flag-XPA WT - - + -
GFP-Flag-XPA 6KR - - - +
XPA shRNA - + + +
(kDa)
75
45
XPA
35
GAPDH
I
Soluble Fraction
GFP-Flag-XPA WT 6KR WT 6KR WT 6KR
NALA - - + + - -
LDHi - - - - + +
UV + + + + + +
(kDa)
75
Flag
35
GAPDH
J
Soluble Fraction
GFP-Flag-XPA WT 6KR WT 6KR WT 6KR
NALA - - + + - -
LDHi - - - - + +
UV + + + + + +
(kDa)
75
Flag
35
GAPDH
K
Soluble Fraction
GFP-Flag-XPA WT 6KR WT 6KR WT 6KR
NALA - - + + + +
MG149 - - - - + +
UV + + + + + +
(kDa)
75
Flag
35
GAPDH
L
Soluble Fraction
GFP-Flag-XPA WT 6KR WT 6KR WT 6KR
NALA - - + + + +
MG149 - - - - + +
UV + + + + + +
(kDa)
75
Flag
35
GAPDH
M
Chromatin Fraction
GFP-Flag-XPA WT WT 6KR 6KR
HA-KAT5 - + - +
UV + + + +
(kDa)
75
Flag
15
H3
Soluble Fraction
GFP-Flag-XPA WT WT 6KR 6KR
HA-KAT5 - + - +
UV + + + +
(kDa)
75
Flag
60
HA
35
GAPDH
N
Chromatin Fraction
GFP-Flag-XPA WT WT 6KR 6KR
HA-KAT5 - + - +
UV + + + +
(kDa)
75
Flag
15
H3
Soluble Fraction
GFP-Flag-XPA WT WT 6KR 6KR
HA-KAT5 - + - +
UV + + + +
(kDa)
75
Flag
60
HA
35
GAPDH
O
Chromatin Fraction
GFP-Flag-XPA WT WT KR KR
UV - + - +
(kDa)
XPG
180
XPF
100
75
Flag
15
H3
Soluble Fraction
GFP-Flag-XPA WT WT KR KR
UV - + - +
(kDa)
XPG
180
XPF
100
75
Flag
35
GAPDH

Figure S12 Lactylation enhances the ability of XPA to bind DNA related to Figure S4

(A-B) Two additional independent replicate experiments for Figure S4A.

(C-D) Two additional independent replicate experiments for Figure S4C.

(E-F) Two additional independent replicate experiments for Figure S4E

(G-H) Two additional independent replicate experiments for Figure S4G.

(I-J) Two additional independent replicate experiments for Figure S4I.

(K-L) Two additional independent replicate experiments for Figure S4K.

(M-N) Two additional independent replicate experiments for Figure S4M.

(O) Additioal independent replicate experiments for Figure S4U.

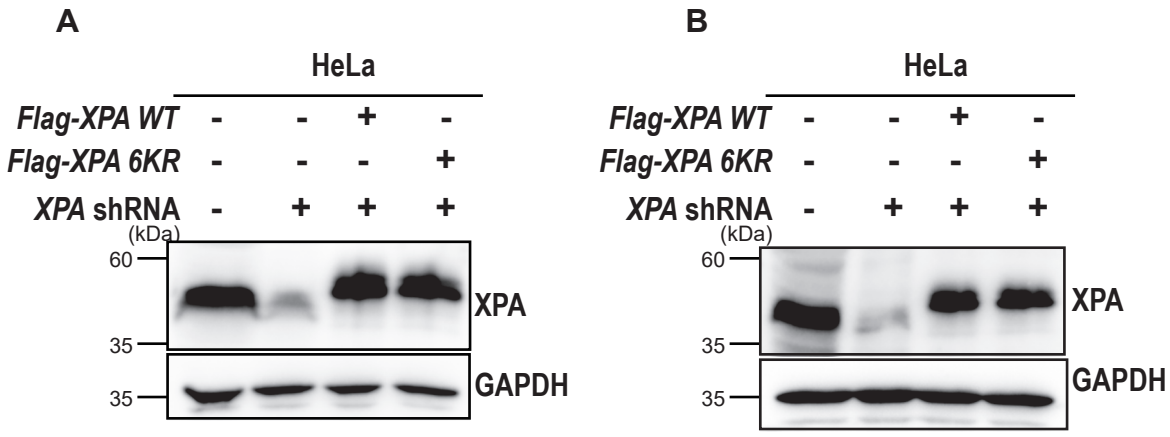

Figure 13 XPA lactylation promotes NER and cell survival under UV-induced damage related to Figure 5

(A-B) Two additional independent replicate experiments for Figure 5A.

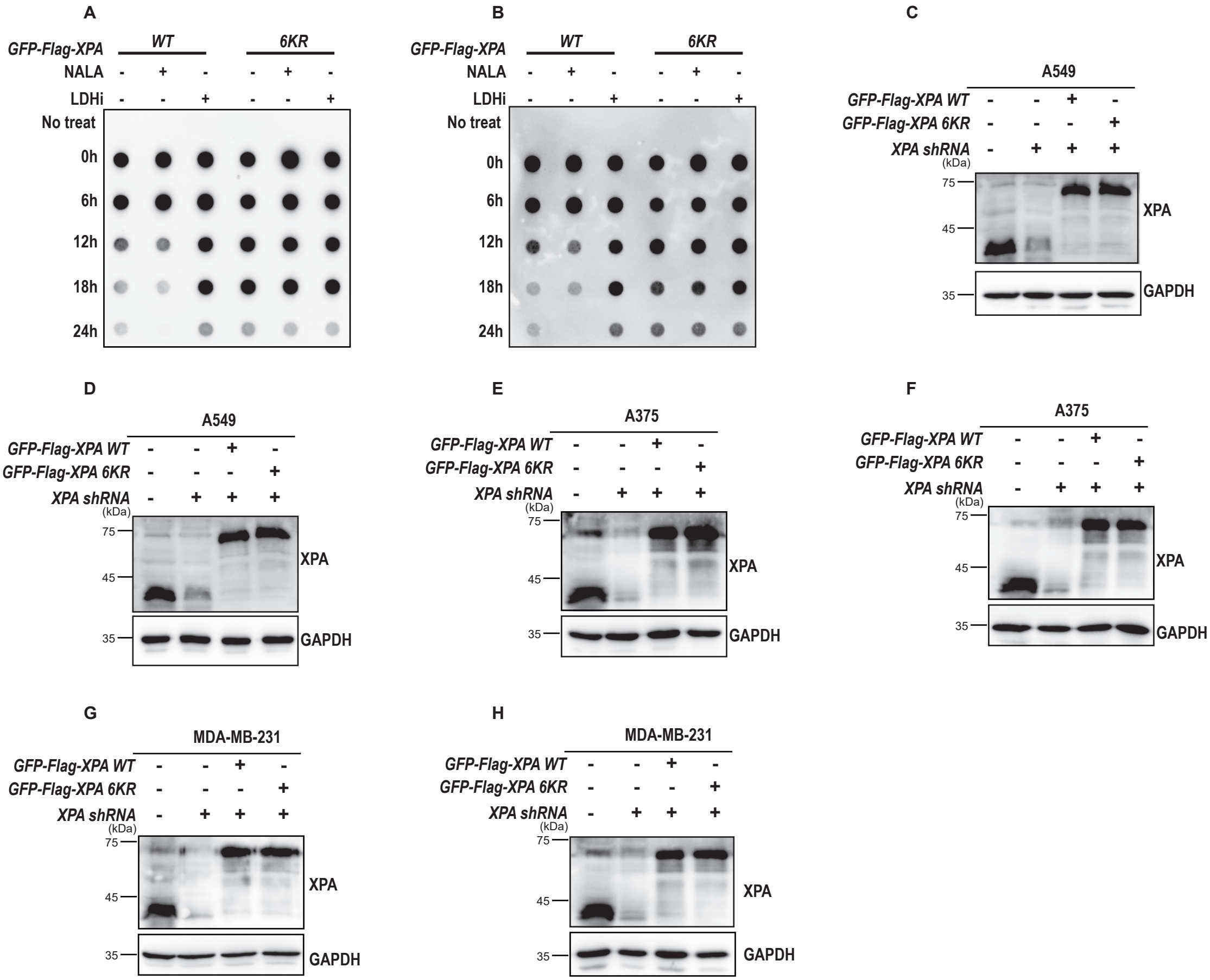

Figure S14 XPA lactylation promotes NER and cell survival under UV-induced damage related to Figure S5

(A-B) Two additional independent replicate experiments for Figure S5A.

(C-D) Two additional independent replicate experiments for Figure S5C.

(E-F) Two additional independent replicate experiments for Figure S5E.

(G-H) Two additional independent replicate experiments for Figure S5G.
